# Unravelling the effects of selective estrogen receptor modulators on colorectal cancer: a prognostic role for insulin-like growth factor binding protein-5

**DOI:** 10.1042/CS20258451

**Published:** 2026-05-21

**Authors:** Xiaoyu Su, Rachel M. Barker, Kathryn McCarthy, Kalina Biernacka, Astor Mak, Claire M. Perks

**Affiliations:** 1 Cancer Endocrinology Group, Bristol Medical School, Learning & Research Building, Level 2, Southmead Hospital, Bristol BS10 5NB, U.K.; 2 North Bristol NHS Trust, Southmead Hospital, Southmead Road, Bristol BS10 5NB, U.K.

**Keywords:** colorectal cancer, estrogen, estrogen receptor β, GPER1, IGFBP-5, obesity

## Abstract

Obesity contributes to colorectal cancer (CRC) by elevating levels of estrogen, 27-hydroxycholesterol (27-OHC), and insulin-like growth factors. Paradoxically, epidemiological studies show that hormone replacement therapy reduces CRC incidence and mortality, particularly in postmenopausal women, suggesting a protective role for estrogen. Estrogen and 27-OHC signal *via* ERα, ERβ, and G protein-coupled estrogen receptor 1 (GPER1), with ERβ being the predominant estrogen receptor in the colon. We observed a strong positive correlation between ERβ and insulin-like growth factor-binding protein-5 (IGFBP-5) in CRC tissues, and high IGFBP-5 expression was significantly associated with poor patient outcomes. Functional assays revealed that ERβ knockdown inhibited colon cancer cell proliferation and migration, accompanied by a marked reduction in IGFBP-5 expression. Co-immunoprecipitation confirmed a direct interaction between ERβ and IGFBP-5. Both estrogen and 27-OHC suppressed CRC cell proliferation and IGFBP-5 expression, but this was independent of ERβ. Combined ERβ silencing and estrogen or 27-OHC treatment enhanced DNA damage and apoptosis. Transcriptome analysis identified GPER1 as a downstream estrogen-responsive gene. Subsequent *in vitro* validation confirmed that GPER1 activation mediates the tumour-suppressive effects of estrogen and 27-OHC. Collectively, our findings highlight IGFBP-5 as a potential prognostic marker, demonstrate the tumour-inhibitory effect of ERβ silencing, and identify GPER1 as a promising therapeutic target linking estrogen signalling, lipid metabolism, and CRC progression.

## Introduction

Colorectal cancer (CRC) is the third most commonly diagnosed cancer and the second leading cause of cancer-related mortality worldwide [1]. From 1999 to 2019, the global incidence of CRC increased significantly, rising from 0.84 million to 2.17 million cases [2]. Despite reductions in new cases in some countries, attributed to healthier lifestyles and early screening initiatives, a notable annual increase of 1%–4% in CRC incidence among individuals under 50 years old has been reported in several nations, including the U.S.A., Canada, and seven other high-income countries [1]. This trend emphasises the significant influence of dietary patterns, excess body weight, and lifestyle factors on the rising incidence of CRC.

The global prevalence of obesity continues to rise, with the World Health Organization reporting that one in eight people worldwide is now classified as obese [3]. Since 1990, the number of obese individuals has surged, particularly among younger populations. Adolescent obesity rates are projected to have quadrupled by 2022 compared with 1990. Obesity is recognised as a significant risk factor for early-onset CRC, and obesity-related metabolic disorders elevate blood levels of estrogen, 27-hydroxycholesterol (27-OHC, the most prevalent oxysterol in the body), and insulin-like growth factors (IGFs) in CRC [1,4].

Estrogen, also known as the female hormone, is a steroidal compound that can be produced by the placenta and ovaries. Natural estrogens include estrone (E1), estradiol (E2), and estriol (E3), and most of the estrogens commonly used in clinical practice are synthetic derivatives of E2 [5–7]. Estrogen exerts its biological effects primarily through binding to specific estrogen receptors (ERs), which include the classical nuclear receptors ERα and ERβ, as well as the G protein-coupled estrogen receptor 1 (GPER1). ERα and ERβ function as ligand-activated transcription factors that regulate gene expression upon binding estrogen [8]. In contrast, GPER1 is a membrane-bound receptor that mediates rapid, non-genomic estrogen signalling via second messenger cascades [9,10]. The diverse expression patterns and functions of these receptors imply the complexity of estrogen signalling in different tissues and disease contexts, including CRC.

Both 27-OHC and E2 function as endogenous selective estrogen receptor modulators (SERMs), binding to ERs to exert their physiological effects [11,12]. Given the predominant expression of ERβ and the extremely low expression of ERα in CRC, most studies have focused on SERMs/ERβ signalling pathway, highlighting the protective role of ERβ in CRC [8,13]. Additionally, ERβ expression is markedly diminished in CRC, and this reduction has been closely linked to the progression of the disease [14]. However, the mechanism of how SERMs/ERβ act in CRC is still unclear. In terms of GPER1, currently, there is no study related to SERMs/GPER1 being reported in CRC.

In addition, insulin-like growth factor-binding protein-5 (IGFBP-5), a member of the IGF family, plays a critical role in modulating IGF activity and has been implicated in the regulation of tumour cell proliferation, invasion, and migration across various cancer types [15]. While IGFBP-5 exerts its classical function by binding to IGFs and regulating their bioavailability, accumulating evidence suggests that it may also influence cellular behaviours through IGF-independent mechanisms [15]. This dual functionality highlights a potential link between ER signalling and the IGF axis in CRC. Notably, knockdown of IGFBP-5 in DLD1 colon cancer cells has been shown to significantly reduce cell migration [16]. Moreover, lower IGFBP-5 expression is associated with decreased cell proliferation and has been correlated with improved patient survival, in contrast with the generally protective role attributed to ERβ in CRC [17,18]. However, whether IGFBP-5 participates in E2 or 27-OHC-mediated regulation of CRC cell behaviour remains largely unexplored.

Given this context, the present study aimed to investigate the effects of estrogen and 27-OHC on the proliferation and migration of CRC cells, with a specific focus on their potential regulation of IGFBP-5 expression through activation of ERβ- or GPER1-related signalling pathways. By systematically elucidating the role of this pathway in CRC, we seek to uncover novel insights into the protective mechanisms of obesity-associated metabolic factors in colorectal carcinogenesis and to identify potential molecular targets for CRC prevention and therapy.

## Materials and methods

### Reagents

Water-soluble 17β-estradiol (E2, Cat. No. E4389) was purchased from Sigma–Aldrich and freshly prepared by dissolving 2 mg in 1 ml of phenol red-free serum-free medium (SFM). The resulting 350 μM stock solution was filtered and further diluted 1:100 to obtain a 3.5 μM working concentration. 27-OHC (Cat. No. SC-358756), a cholesterol metabolite soluble in ethanol, was obtained from Santa Cruz Biotechnology. A 1 mM stock solution was prepared in absolute ethanol and stored at –70°C until use. G1, a potent and selective GPER1 agonist (Cat. No. HY-107216), was obtained from MedChemExpress and dissolved in 100% dimethyl sulfoxide at 1 mM for storage at –30°C. All compounds were handled in accordance with the manufacturers' protocols and diluted in appropriate vehicles for experimental use.

### Cell culture

Four human CRC cell lines (HCT-116, SW620, HT-29, HCA-7) and one normal colonic epithelial cell line (FHC) were utilised in the present study. HCT-116, SW620, HT-29, and FHC were sourced from the American Type Culture Collection, while HCA-7 was obtained from the European Collection of Authenticated Cell Cultures. FHC cells were cultured in DMEM/F12 medium supplemented with 10% fetal bovine serum (FBS), 1% L-glutamine, 0.005 mg/ml insulin, 0.005 mg/ml apo-transferrin, 100 ng/ml hydrocortisone, 20 ng/ml recombinant human EGF, 10 ng/ml cholera toxin, and 10 mM HEPES. HCT-116 and HT-29 were maintained in McCoy's 5A medium with 10% FBS and 1% L-glutamine, whereas SW620 and HCA-7 were cultured in high-glucose DMEM supplemented similarly. All cultures were maintained at 37°C in a humidified incubator with 5% CO_2_. Authentication was confirmed via STR profiling, and all cell lines were verified as mycoplasma-free.

### Tritiated thymidine incorporation assay

To evaluate DNA synthesis, a tritiated thymidine incorporation assay was conducted. 2.5 × 10^4^ cells were seeded into 24-well plates and cultured for 24 h before medium was switched to serum-free, phenol red-free DMEM supplemented with 1% L-glutamine, 10 mg/l apo-transferrin, 1.2 g/l sodium bicarbonate, and 0.2% BSA. After 48 h of treatment, [^3^H]-thymidine was added for 4 h. Subsequently, cells were fixed with ice-cold 5% trichloroacetic acid and lysed with 1M NaOH. Radioactive incorporation was measured using a scintillation counter (LS6500, Beckman), and results were expressed as disintegrations per minute.

### RNA interference

Gene knockdown was performed using siRNA targeting ERβ. HCT-116 and HT-29 cells (3 × 10^5^ cells/well) were seeded into six-well plates and transfected with 50 nM siRNA (Dharmacon, ON-TARGET plus Human ESR2 (2100) siRNA-SMART pool, 20 nmol (L-003402-00-0020)) as performed previously [19] using RNAiMAX Lipofectamine (Invitrogen) in phenol red-free Opti-MEM medium. Non-targeting siRNA (Qiagen) served as a negative control. After 24 h of transfection, RNA and protein were harvested on days three and four for downstream analyses, respectively. The ERβ sequences used for siRNA were shown below: J-003402-13 GGAAAUGCGUAGAAGGAAU, J-003402-14 UUCAAGGUUUCGAGAGUUA, J-003402-15 GCACGGCUCCAUAUACAUA, J-003402-16 GAACCCACAGUCUCAGUGA.

### Holographic imaging for proliferation and migration

A digital holographic imaging platform (HoloMonitor) was employed for real-time, label-free analysis of cell proliferation and motility. Colon cell lines (FHC, HCT-116, SW620, HT-29) were seeded at 1.5 × 10^4^ cells/well in 24-well plates as performed previously [20]. After serum starvation, cells were treated with varying concentrations of E2 (0.625–5 nM) or 27-OHC (0.001–10 μM). In a separate wound healing setup, HCT-116 cells were seeded into silicone inserts (ibidi), transfected with ERβ siRNA, and the insert was removed after 24 h for migration tracking. Data acquisition and quantitative analysis were performed using HoloMonitor App Suite software.

### Transwell migration assay

Transwell chambers (8 μm pore size, Greiner Bio-One) were used to evaluate directional cell migration. CRC cell lines (0.3 × 10^6^/T25 flask) were pre-treated with E2 or 27-OHC for 48 h in phenol red-free SFM, and then 4 × 10^5^ cells were reseeded in the upper chamber in SFM. After 16–24 h, migrated cells were fixed with 4% paraformaldehyde, permeabilised with 0.2% Triton X-100, and stained with 0.05% crystal violet. The dye was eluted using 1% SDS, and absorbance was read at 595 nm (iMark plate reader, BioRad). The assay protocol was adapted from previously validated procedures [19].

### TCGA dataset analysis and CCLE dataset analysis

Transcriptomic and survival data were analysed using GEPIA2 and a custom pipeline in R (v4.3.3) [21]. While GEPIA2 enabled visualization of baseline mRNA expression in colorectal cohorts (total CRC *n* = 342; colon cancer *n* = 254; rectal cancer *n* = 88), a curated TCGA (The Cancer Genome Atlas) dataset (total *n* = 526) was used for in-depth correlation and survival analysis. Gene–gene associations were calculated using the ggstatsplot package, and heatmaps were generated with pheatmap. Kaplan–Meier survival curves were constructed with ggplot2 and survival packages. Optimal cut-offs were determined via the Human Protein Atlas algorithm (https://www.proteinatlas.org/). CRC cell line gene expression data were acquired from the Cancer Cell Line Encyclopedia (CCLE). Log2(TPM + 1) expression levels for ESR1 and ESR2 were obtained from CRC cell lines. Data visualisation was produced *via* the ggplot2 and ggpubr packages in R.

### Quantitative real-time PCR

Total RNA from cells seeded at 0.3 × 10^6^ was isolated using TRIzol reagent (Thermo Fisher) as previously described [22]. Primer sequences are listed in Supplementary Table S1. Gene expression was normalised to GAPDH, and fold changes were computed by the ΔΔCt method [23]. All reactions were performed in triplicate.

### Immunofluorescence microscopy

For immunocytochemistry, cells were grown on glass coverslips and fixed with 4% paraformaldehyde as described before [24]. In brief, fixed cells were quenched with 300 mM glycine, permeabilised with 0.5% Triton X-100, and blocked with 5% normal goat serum. Primary antibodies were applied overnight at 4°C, followed by fluorophore-conjugated secondary antibodies for 1 h. Nuclear staining was achieved with DAPI-containing Vectashield mounting medium. Images were acquired using a Leica SP5 confocal microscope and analysed with FIJI/ImageJ software. Antibody details are provided in Supplementary Table S2.

### Immunoprecipitation

To examine protein–protein interactions, 500 μg of HCT-116 cell lysates were incubated with 2 μg of anti-ERβ (Invitrogen, PPZ0506) antibody or isotype IgG (negative control) overnight at 4°C as performed previously [25]. A/G Plus agarose beads (Santa Cruz) were added the following day for 2 h. After washing, immune complexes were eluted with 2× Laemmli buffer and heated at 95°C for 10 min before analysis by Western blot.

### Western blotting

Cell lysates were prepared in RIPA buffer supplemented with protease/phosphatase inhibitors. Protein concentrations were quantified using a BCA assay. Equal amounts (30–40 μg) were resolved on 4%–20% SDS-PAGE gels and transferred to nitrocellulose membranes. After blocking in 5% semi-skimmed milk–TBST, membranes were probed with primary antibodies overnight, followed by HRP-conjugated secondary antibodies. Signals were detected using enhanced chemiluminescence and visualised with a Bio-Rad ChemiDoc system. Densitometry was performed using Image Lab software. Antibody details are provided in Supplementary Table S2.

### Protein–protein docking analysis

Molecular docking was conducted using the ZDOCK 3.0.2 platform to predict potential binding interfaces between IGFBP-5 (PDB: 1H59) and ESR2 (PDB: 3OLL) [26]. This algorithm incorporates rigid-body docking based on shape complementarity and electrostatics. Top-ranked docking models were visualised in PyMOL (The PyMOL Molecular Graphics System, Version 3.0.0, Schrödinger, LLC), and interaction interfaces were identified by residue proximity within 3 Å. Detailed residue interactions and structural illustrations are provided in Supplementary Table S3.

### Calcium quantification assay

To assess intracellular calcium levels, a colorimetric calcium assay kit (Abcam, ab102505) was used. Colon cancer cells (HCT-116 and HT-29) were treated with 10 nM E2 and 0.1 μM, 1 μM 27-OHC for 15 min and then 1 × 10^6^ cells per group were counted and lysed in 200 μl of cell lysis buffer. After cell lysis in calcium assay buffer, samples were centrifuged, and supernatants were assayed alongside standards. Each 96-well plate well received 50 μl of sample or standard, followed by 90 μl of chromogenic reagent and 60 μl of assay buffer. Absorbance was recorded at 575 nm using a microplate reader.

### cAMP enzyme immunoassay

Cyclic adenosine monophosphate (cAMP) levels were measured using a competitive ELISA kit (Screen Quest™). The assay uses HRP-conjugated cAMP and anti-cAMP antibody-coated plates. Colon cancer cells (HCT-116 and HT-29) were treated with 10 nM E2 and 0.1 μM, 1 μM 27-OHC for 15 min and then 1 × 10^6^ cells per group were counted and lysed in 200 μl of cell lysis buffer for immune reaction. After incubation with standards and samples, Amplite™ Green substrate was added. The absorbance, inversely proportional to cAMP levels, was measured at multiple wavelengths. Data were analysed using a four-parameter logistic curve-fitting tool (https://www.aatbio.com/tools/four-parameter-logistic-4pl-curve-regression-online-calculator).

### Analysis of public transcriptomic datasets

RNA-seq data from the GEO dataset GSE112568, which profiles SW620 colon cancer cells treated with G1 (1 μM) for 24 h, were analysed using DESeq2. Differentially expressed genes (DEGs) were identified using a threshold of |Log2FC| ≥1 and adjusted *P* <0.05. Functional enrichment of DEGs was performed via GO analysis, with visualization using ggplot2 in R (Supplementary Excel Data S1).

### Statistical analysis

All experimental data are presented as mean ± standard deviation (SD) or mean ± standard error, as appropriate. Statistical comparisons between two groups were made using unpaired Student's *t*-tests, while multiple group comparisons were assessed using one-way ANOVA followed by Dunnett or Tukey *post hoc* tests. All analyses were performed using GraphPad Prism 10.2, with statistical significance defined as *P* <0.05 (*), *P* <0.01 (**), and *P* <0.001 (***).

## Results

### Expression patterns of ERα and ERβ in colon cancer

Previous studies have suggested that ERβ expression is markedly reduced in CRC, whereas ERα is expressed at very low levels. However, a systematic comparison of ERα and ERβ expression across different CRC cell lines remains limited.

**Figure 1:**
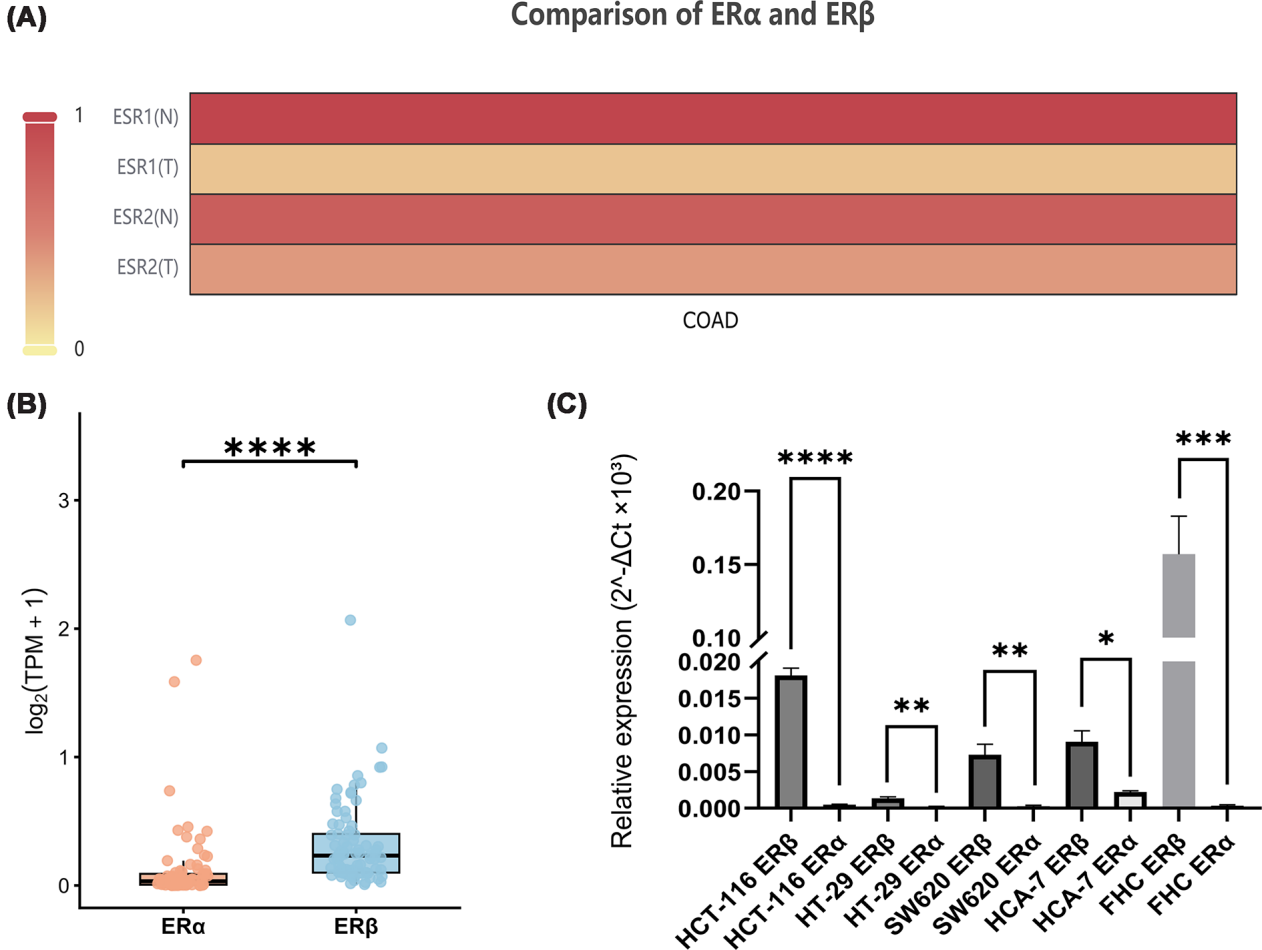
Differential expression of ERα and ERβ in colon cancer. (**A**) Comparison of ESR2 (ERβ) and ESR1 (ERα) in colon cancer (colon adenocarcinoma (COAD)) using the GEPIA2 database. (**B**) Comparison of basal abundance between ERα and ERβ in COAD cell lines through CCLE database. (**C**) Relative mRNA levels of ERα and ERβ across five cell lines normalised to FHC cells. Data were expressed as 2^−ΔCt^ values (multiplied by 10^3^ for visualization), which represents the ratio of target mRNA to *GAPDH* mRNA. All data are presented as mean ± SD of three independent biological replicates. Statistical significance was determined by two-way ANOVA.

To address this, we first analysed RNA-seq data from the TCGA cohort. As shown in Figure 1A, both ERα and ERβ expression levels were significantly lower in colon cancer tissues compared with normal colon tissues (*P* <0.0005). Analysis of the CCLE dataset further supported these observations. Across 82 colon cancer cell lines, ERβ expression was consistently higher than ERα expression (Figure 1B). Our experimental data showed the same pattern (Figure 1C and Supplementary Figure S1), with ERβ levels significantly exceeding those of ERα in both normal colon epithelial cells and colon cancer cell lines. In contrast, ERα expression was extremely low and in some cases close to the detection limit. To indicate further that ERα does not play a major role in this context, we found that ERα remained negligible when ERβ was silenced (data not shown).

Taken together, evidence from TCGA datasets, CCLE data, and our experimental results consistently indicates that ERβ is the dominant ER in colorectal tissues, whereas ERα expression is minimal. Therefore, the biological effects of estrogen in CRC are more likely to be mediated primarily through ERβ rather than ERα. Based on these findings, subsequent functional experiments were designed to primarily investigate the role of ERβ in mediating the effects of estrogen in colon cancer cells.

### Anti-proliferative effects of 27-OHC and E2 in colon cancer cell lines

**Figure 2:**
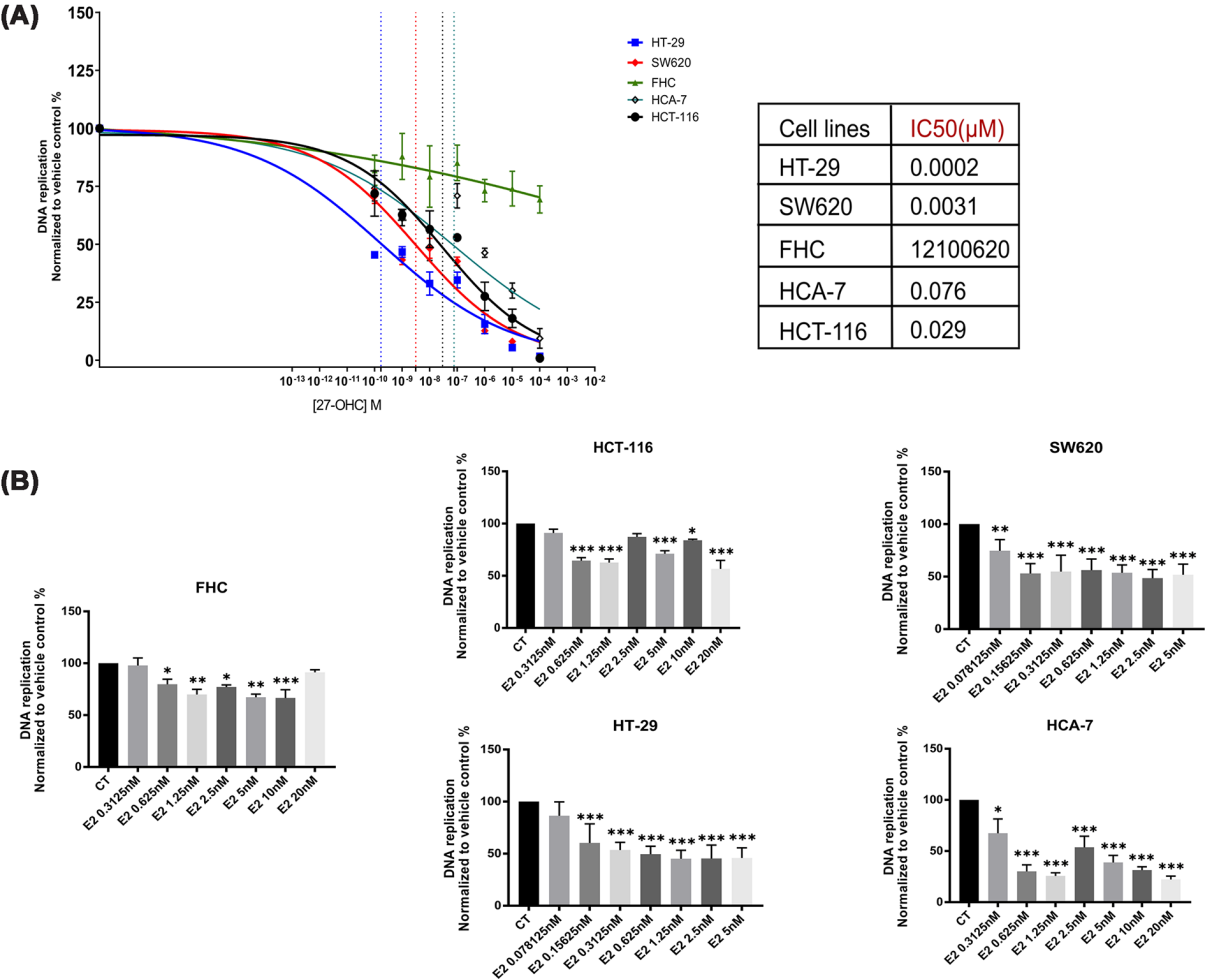
Anti-proliferative effects of 27-OHC and E2 in colon cancer cell lines. (**A**) Effects of 27-OHC on cell proliferation after 48 h of treatment across five colon cell lines. The assay was performed in triplicate and repeated in three independent biological experiments (*n* = 3). The figure presents the dose-dependent impact of 27-OHC on the replicative capacity of each cell line. FHC (normal colonic epithelial cells) is represented in green, HCT-116 in black, SW620 in red, HT-29 in blue, and HCA-7 in olive green. The *x*-axis indicates the concentration of 27-OHC, while the *y*-axis shows the relative DNA replication capacity, normalised to the vehicle-treated control group. Corresponding statistical analyses are provided in Supplementary Figure S2. (**B**) Inhibitory effects of E2 on cell proliferation after 48 h of treatment in five colon cell lines. Experimental procedures were performed in triplicate and repeated in three independent biological replicates (*n* = 3). The *x*-axis shows the range of E2 concentrations tested, and the *y*-axis reflects normalised cellular proliferative capacity relative to the control group. Data are presented as mean ± SD.

Previous studies have suggested that both E2 and 27-OHC may influence colon cancer cell proliferation. However, most published studies have examined only one or two cell lines under different experimental conditions. To obtain a more systematic assessment, we evaluated the effects of E2 and 27-OHC across four colon cancer cell lines derived from both male and female donors and one normal colon cell line. As illustrated in Figure 2A (and Supplementary Figure S2), treatment with 27-OHC significantly reduced cell proliferation in all four colon cancer cell lines tested, exhibiting a clear concentration-dependent pattern. Among these, HT-29 cells—originating from a female donor—displayed the highest sensitivity to 27-OHC, with an IC50 value of 0.00017 μM. Conversely, the HCA-7 cell line, also female-derived, was the least sensitive, showing an IC50 of 0.076 μM. For the male-derived cell lines, SW620 was more responsive to 27-OHC (IC50 = 0.0032 μM) compared with HCT-116 (IC50 = 0.029 μM). The normal colon epithelial cell line FHC exhibited minimal inhibition upon 27-OHC exposure, likely due to high variability among replicates, as reflected by a large standard error, suggesting a less robust or consistent response in non-malignant cells.

In parallel, E2 also demonstrated growth-inhibitory effects across all colon cancer cell lines (Figure 2B), though this response was less clearly dose-dependent. Notably, both HT-29 and SW620 cells exhibited marked reductions in proliferation at lower E2 concentrations, with up to 50% inhibition observed between 0 and 0.156 nM. However, increasing E2 beyond this level did not produce further significant decreases in cell growth. A comparable trend was seen in SW620, reinforcing its sensitivity to low-dose E2.

In contrast, HCT-116 cells showed inconsistent proliferation patterns in response to E2. While 20 nM E2 resulted in a 50% inhibition, intermediate concentrations yielded variable outcomes; for instance, 0.3125–1.25 nM reduced proliferation, whereas 2.5 nM showed no significant effect. Similarly, although HCA-7 cells responded more strongly at 0.625 nM, their sensitivity diminished at higher doses.

Interestingly, E2 also significantly suppressed proliferation in FHC cells at concentrations between 0.625 and 10 nM, though the overall sensitivity was lower than that observed in cancer cells. No further reduction was detected at 20 nM compared with the untreated control.

Collectively, these findings confirm the anti-proliferative properties of both 27-OHC and E2 in colon cancer cells, with 27-OHC showing consistent, concentration-dependent effects. E2 responses varied by cell line and concentration, without consistent sex-related differences, highlighting the need for dose optimization in future mechanistic studies. Importantly, by analysing multiple CRC cell lines under comparable experimental conditions, our findings provide a more comprehensive overview of the variability in cellular responses to these compounds.

### Inhibitory effects of 27-OHC and E2 on cell motility in colon cancer cell lines

To further explore the cellular effects of 27-OHC and E2 beyond proliferation, we assessed their impact on cell motility using live-cell quantitative phase imaging with a HoloMonitor live-cell imaging system. This platform allows continuous tracking of individual cell movement and provides quantitative parameters such as cell speed and accumulated migration distance.

**Figure 3:**
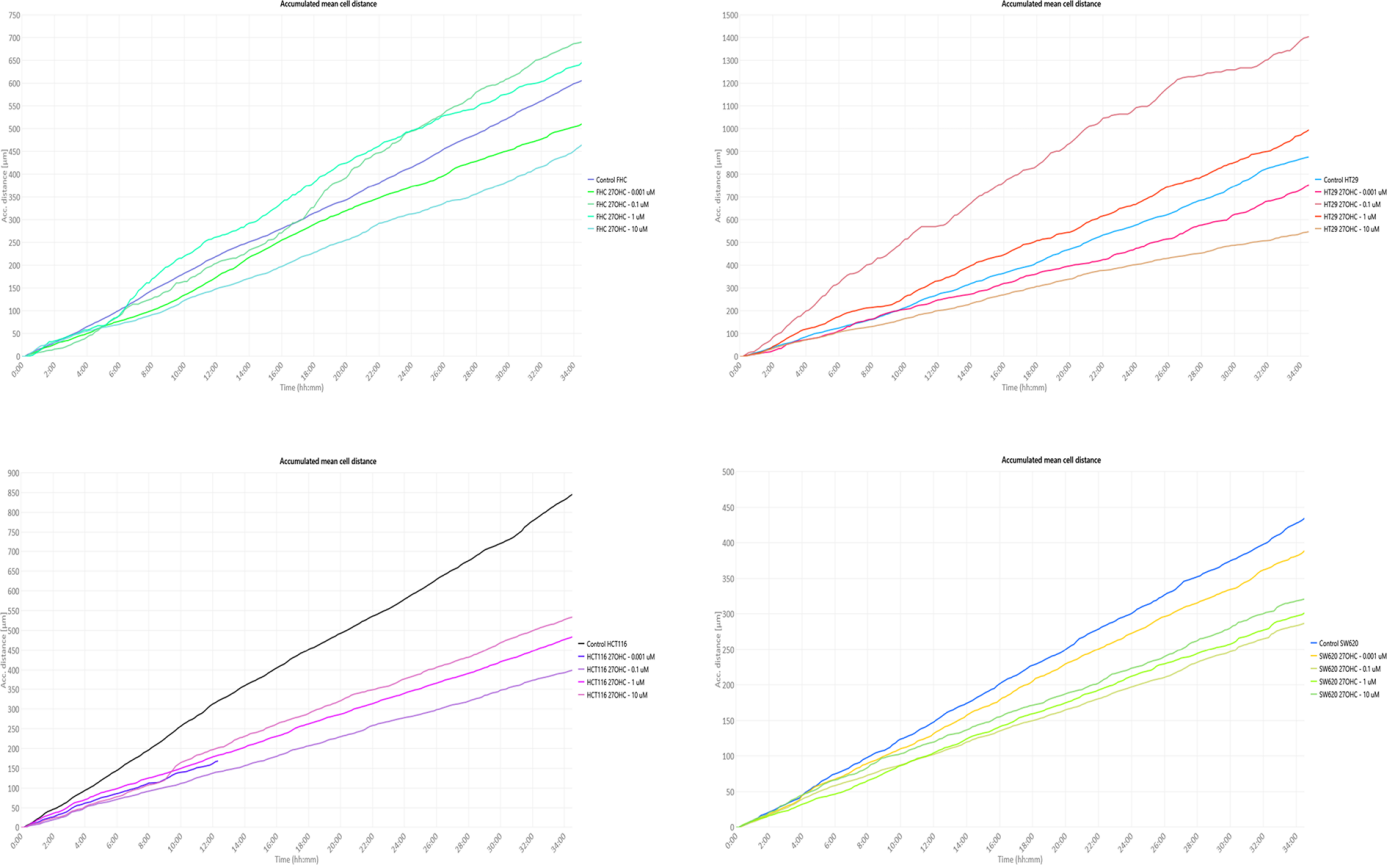
Effects of 27-OHC on cell motility in colon cell lines. This figure depicts the motility responses of four colon cell lines—HCT-116, SW620, HT-29, and FHC—following 48 h of treatment with 27-OHC. Cell motility was quantitatively analysed using the HoloMonitor live-cell imaging assay. Based on previously determined IC_50_ values from the thymidine incorporation assay (Figure 2), four concentrations of 27-OHC (0.001, 0.1, 1, and 10 μM) were selected for this experiment. The *x*-axis indicates the time intervals across the 48-h observation period, while the *y*-axis shows the average displacement of cells from their original positions, reflecting dynamic motility behaviour. The results reveal cell-type-specific variations in motility in response to different doses of 27-OHC.

Analysis of time-lapse recordings revealed that 27-OHC reduced the motility of HCT-116 and SW620 cells in a concentration-dependent manner (Figure 3). Higher concentrations were associated with lower mean cell speeds and shorter accumulated movement distances over the observation period. In contrast, HT-29 cells displayed a more variable response, with minor increases in motility observed at lower concentrations, while higher doses resulted in reduced movement. The normal colon epithelial cell line FHC exhibited a similar trend.

**Figure 4:**
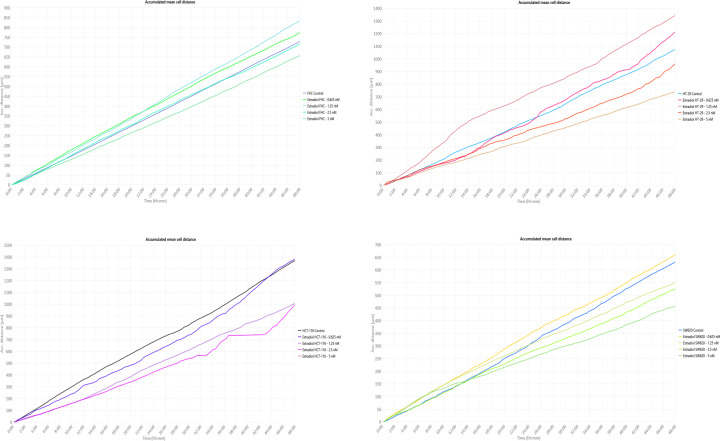
Effects of E2 on cell motility in colon cell lines. This panel illustrates the motility responses of the same four colon cell lines (HCT-116, SW620, HT-29, and FHC) after 48 h of treatment with E2, also assessed using the HoloMonitor platform. Concentrations of E2 (0.625, 1.25, 2.5, and 5 nM) were selected based on cell proliferation results from earlier assays (Figure 2). Similar to Figure 3, the *x*-axis represents time intervals, while the *y*-axis reflects changes in cell displacement relative to their starting positions. The data demonstrate a biphasic or inhibitory pattern in motility, depending on concentration and cell line.

E2 treatment produced broadly comparable effects (Figure 4). In HCT-116 and SW620 cells, increasing E2 concentrations was generally associated with reduced cell motility parameters. HT-29 cells again showed more heterogeneous responses, with modest increases in movement observed at lower concentrations, followed by reduced motility at higher doses.

**Figure 5:**
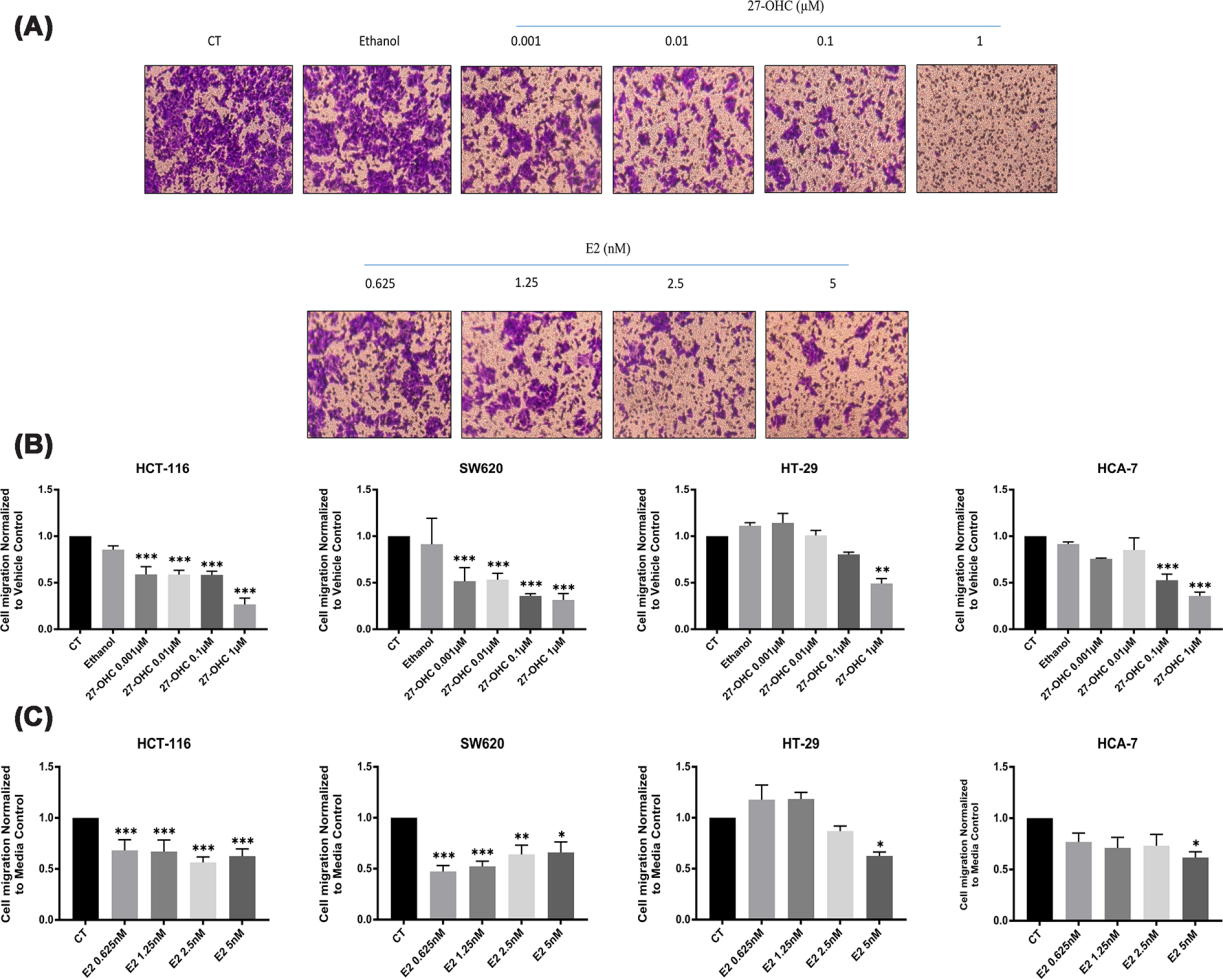
Effects of 27-OHC and E2 on colon cancer cell migration. (**A**) Representative microscopic images showing the dose-dependent effects of 27-OHC and E2 on cell migration in HCT-116 cells after 48 h of treatment. Cells were exposed to four concentrations of 27-OHC (0.001, 0.01, 0.1, and 1 μM) and E2 (0.625, 1.25, 2.5, and 5 nM). Images were captured prior to the application of 1% SDS and reflect differences in cell migration across doses. (**B**) Quantitative analysis of 27-OHC-induced migration changes across four colon cancer cell lines. Cell migration was assessed using a trans-well assay following 48-h treatment with 27-OHC at the indicated concentrations. Experiments were performed in triplicate across three biological replicates (*n* = 3). (**C**) Quantitative evaluation of E2-induced migration responses in colon cancer cells. Similarly, cell migration was assessed after 48 h of E2 treatment using the same four concentrations. Data were normalised to phenol red-free SFM controls and presented as fold changes relative to the untreated group. All graphs represent the mean ± SD. Statistical significance was determined using one-way ANOVA followed by Dunnett's *post hoc* test.

To further examine directional cell migration, we performed transwell migration assays following hormone treatment (Figure 5). Both 27-OHC and E2 were associated with reduced numbers of migrating HCT-116 and SW620 cells. For HT-29 and HCA-7, lower concentrations had minimal impact, whereas doses ≥1 μM induced a marked reduction. However, because these experiments were conducted following a 48-h treatment period, the reduced migration observed may partially reflect the anti-proliferative effects of these compounds rather than a direct inhibition of migratory capacity.

Taken together, these findings indicate that both 27-OHC and E2 influence the dynamic behaviour of colon cancer cells, affecting parameters related to cellular movement. However, further experiments specifically designed to distinguish proliferation from migration effects would be required to clarify the underlying mechanisms.

### The effects of 27-OHC and E2 on ERβ and IGFBP-5 expression in colon cancer cell

Previous studies have demonstrated that both 27-OHC and E2 can suppress cell proliferation and migration in colon cancer cells [27–29]. To further investigate the molecular mechanisms underlying these effects, we examined whether the ERβ serves as a downstream target of 27-OHC and E2 in cancer cells, and whether IGFBP-5, a known modulator of cancer progression, is also affected by these treatments [19,30–34]. Protein expression levels of ERβ and IGFBP-5 were evaluated by Western blot analysis following 48 h of treatment.

**Figure 6:**
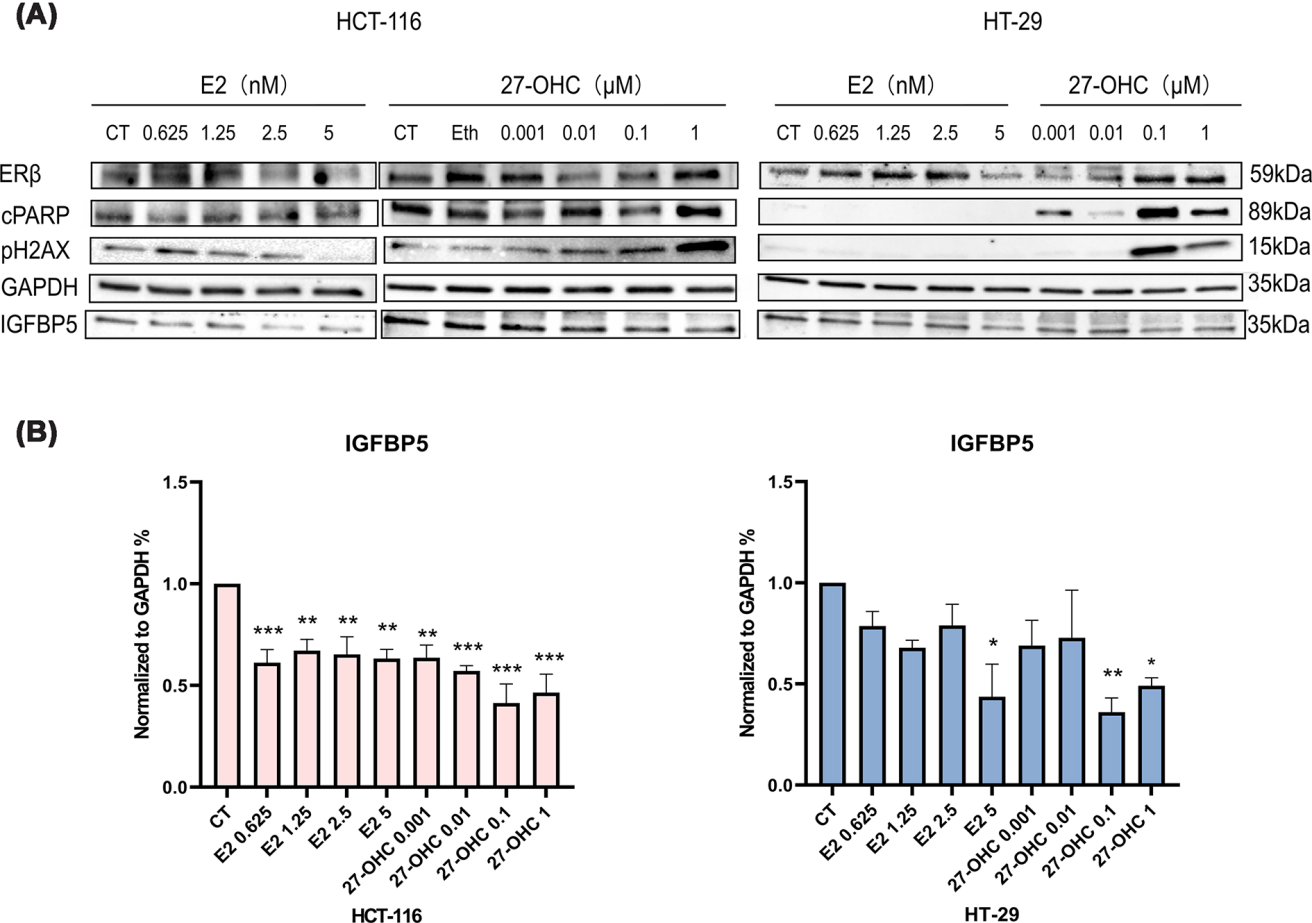
Effects of 27-OHC and E2 on ERβ and IGFBP-5 expression in colon cancer cells. Panels (**A**, **B**) illustrate the effect of varying concentrations of 27-OHC and E2 on the abundance of ERβ and IGFBP-5 in HCT-116 and HT-29 cell lines. The experiment was conducted in three biological repeats (*n* = 3), with four different concentrations of 27-OHC (0.001, 0.1, 1, and 10 μM) or E2 (0.625, 1.25, 2.5, and 5 nM) used for treatment. The raw data from each Western blot experimental group were analysed through Image J and normalised against the phenol red-free SFM control group and were presented as fold changes relative to the control. Statistical analysis of differences was conducted using a one-way ANOVA with Dunnett's *post hoc* test showing with mean ± SD.

As illustrated in Figure 6A, no consistent or reproducible alterations in ERβ protein levels were observed in HCT-116 or HT-29 cell lines following treatment with either 27-OHC or E2. While a modest increase in ERβ expression was occasionally observed in HT-29 cells treated with 27-OHC, the results lacked reproducibility across biological replicates (data from all three experiments not shown), indicating that the regulation of ERβ by these compounds may be minimal or context-dependent.

In contrast, both 27-OHC and E2 consistently suppressed the expression of IGFBP-5 in the HCT-116 and HT-29 cell lines (Figure 6B). In HCT-116 cells, IGFBP-5 protein levels were significantly reduced across all tested concentrations, with no clear dose–response relationship observed, highlighting a higher sensitivity of these male-derived cells to both agents. In HT-29 cells, a similar trend was observed; however, statistically significant reductions in IGFBP-5 were only evident at the higher concentrations of 27-OHC (1 and 10 μM) and E2 (5 nM), suggesting a relatively attenuated response compared with HCT-116 cells.

In addition, treatment with higher concentrations of 27-OHC was associated with increased expression of cleaved poly(ADP-ribose) polymerase (cPARP) and phosphorylated histone H2AX (pH2AX), markers of apoptosis and DNA damage, respectively (Figure 6A). These effects were primarily observed at concentrations ≥1 μM, suggesting that high-dose 27-OHC may induce cellular stress responses in colon cancer cells. Importantly, as these markers reflect apoptotic and DNA damage-related processes, it cannot be excluded that the observed reduction in IGFBP-5 expression under certain conditions may, at least in part, be influenced by decreased cell viability rather than direct regulation. Notably, IGFBP-5 reduction was also detectable under conditions where apoptosis markers were not prominently induced, suggesting that this effect may not be solely attributable to apoptosis.

Collectively, these findings indicate that while the regulation of the levels of ERβ by 27-OHC and E2 remains inconsistent, both compounds are associated with decreased IGFBP-5 protein levels in colon cancer cells, particularly in male-derived colon cancer cells. Further studies will be required to determine whether IGFBP-5 is directly regulated by ERβ signalling or indirectly affected by treatment-induced cellular stress.

### The relationship between ERβ and IGFBP-5 expression following 27-OHC and E2 treatment in colon cancer

Previous literature has identified ERβ as a critical mediator of 27-OHC and E2 in regulating CRC development [35,36]. However, our study did not reveal consistent modulation of ERβ protein levels following treatment with 27-OHC or E2 in HCT-116 or HT-29 colon cancer cell lines. To further investigate the potential involvement of ERβ in the anti-proliferative actions of 27-OHC and E2, we silenced ERβ expression using siRNA in both cell lines and evaluated the resulting cellular responses.

**Figure 7:**
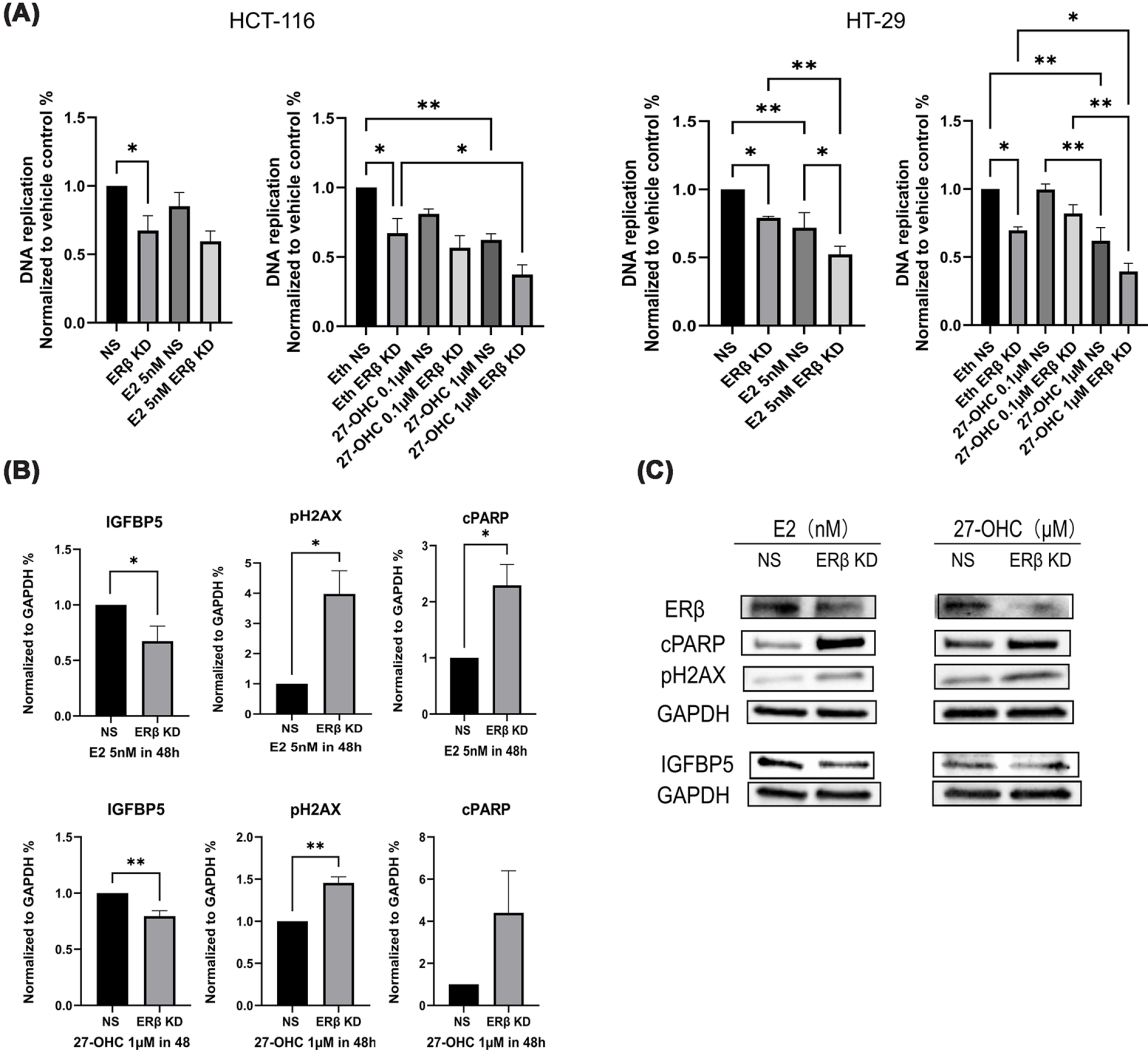
Effects of ERβ silencing on IGFBP-5-associated responses following 27-OHC and E2 treatment in colon cancer cells. (**A**) Proliferative responses of ERβ-silenced HCT-116 and HT-29 cells following 48-h treatment with 27-OHC (0.1and 1 μM) or E2 (5 nM). The experiment was conducted in three independent biological replicates, each performed in triplicate (*n* = 3). Data are presented as mean ± SD and statistical analysis was performed using one-way ANOVA with Tukey multiple comparisons test. (**B**,** C**) Quantification of IGFBP-5, cPARP, and pH2AX protein levels in HCT-116 cells following 48-h treatment with 1 μM 27-OHC or 5 nM E2 in the presence or absence of ERβ knockdown. Protein expression was assessed by Western blotting. Densitometric analysis was performed using ImageJ software, and results are expressed as mean ± SD from three independent biological replicates (*n* = 3), each carried out in triplicate. Statistical significance was evaluated using Student's *t*-test.

As shown in Figure 7A, ERβ knockdown alone significantly reduced the proliferation capacity of both HCT-116 and HT-29 cells. When ERβ silencing was combined with E2 treatment, the HT-29 cell line exhibited a more pronounced suppression of proliferation than either intervention alone. However, this combinatorial effect did not exceed the expected additive inhibition, suggesting that the two treatments may not act synergistically. A similar trend was observed in HCT-116 cells, where the combined treatment of ERβ knockdown and 5 nM E2 did not further enhance the anti-proliferative effect beyond that of E2 or ERβ silencing alone. Parallel results were obtained when cells were treated with 1 μM 27-OHC, indicating a possible antagonistic or overlapping mechanism. These findings imply that ERβ-independent pathways may contribute to the anti-tumour effects of 27-OHC and E2.

To clarify whether IGFBP-5 expression is regulated through ERβ, ERβ was silenced in HCT-116 cells, followed by exposure to 1 μM 27-OHC or 5 nM E2 for 48 h. As illustrated in Figure 7B,C, both treatments led to a further reduction in IGFBP-5 protein levels under ERβ-deficient conditions, suggesting that ERβ may be involved in maintaining IGFBP-5 expression. Additionally, ERβ silencing significantly enhanced the expression of pH2AX, particularly in cells treated with 27-OHC. Although E2 alone did not induce notable changes in apoptotic markers such as cPARP or pH2AX (Figure 7B,C), their expression was markedly elevated following ERβ knockdown in the presence of E2, suggesting that E2 can promote apoptosis and DNA damage in the absence of ERβ-mediated signalling. However, as these markers reflect cellular stress responses, it cannot be excluded that such changes may also contribute to the observed reduction in IGFBP-5 expression under these conditions.

In conclusion, ERβ may be involved in the regulation of IGFBP-5 expression in colon cancer cells, and its depletion sensitizes cells to DNA damage and apoptosis induced by 27-OHC and E2. These results underscore a potentially protective role of ERβ in modulating cellular stress responses and further support a potential link between ERβ signalling and IGFBP-5 expression.

### The interaction between ERβ and IGFBP-5 in colon cancer

**Figure 8:**
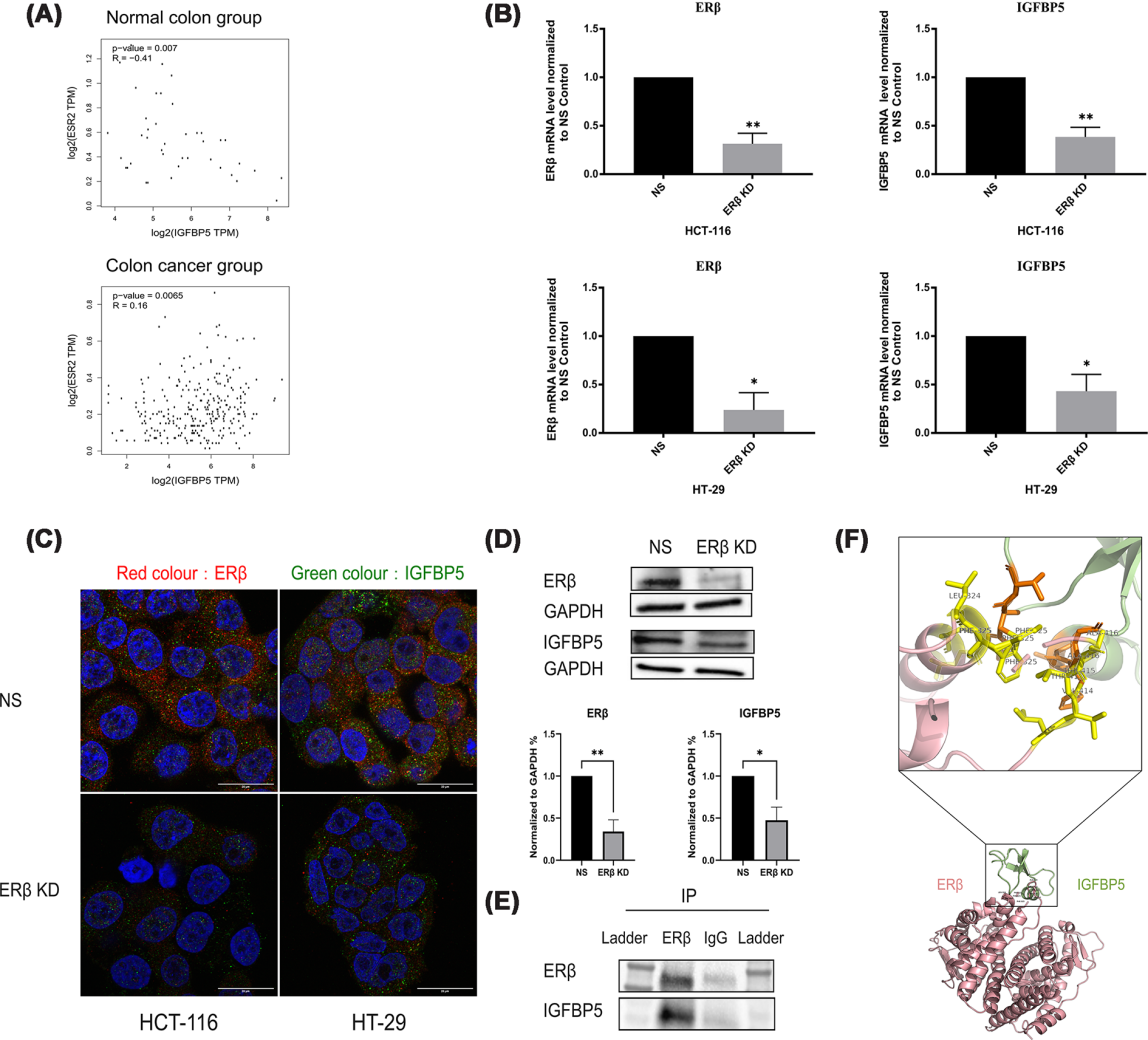
Functional association between ERβ and IGFBP-5 in colon cancer cells. (**A**) Pearson correlation analysis between ERβ and IGFBP-5 mRNA expression levels in colon cancer and normal tissues using the GEPIA2 database. The *x*-axis represents IGFBP-5 mRNA expression, and the *y*-axis represents ERβ mRNA expression. A positive correlation suggests simultaneous increases or decreases in expression of both genes, whereas a negative correlation indicates inverse expression trends. The correlation coefficient (*R*) ranges from −1 to 1, with values closer to ±1 indicating a stronger correlation. A significant positive correlation was observed in colon cancer tissues, whereas a significant negative correlation was found in normal tissues. No statistically significant correlation was noted in rectal cancer tissues. (**B**) Relative mRNA expression of IGFBP-5 following ERβ silencing in HCT-116 and HT-29 colon cancer cell lines. The experiment was conducted in three independent biological replicates (*n* = 3), and results are presented as mean ± SD. Statistical analysis was performed using Student's *t*-test. (**C**) Confocal immunofluorescence images showing the subcellular localization of ERβ (red) and IGFBP-5 (green) in HCT-116 and HT-29 cells, with nuclei counterstained using DAPI (blue). ERβ silencing visibly reduced IGFBP-5 signal intensity, supporting their regulatory association at the protein level. (**D**) Western blot analysis of IGFBP-5 protein expression in HCT-116 cells following ERβ knockdown. Densitometric quantification confirmed the down-regulation of IGFBP-5 protein in response to ERβ silencing. (**E**) Co-immunoprecipitation assay demonstrating the association between ERβ and IGFBP-5 in HCT-116 cells. ERβ was immunoprecipitated, and IGFBP-5 was detected in the precipitated complex by Western blotting, suggesting a potential physical interaction. (**F**) Protein–protein docking model of ERβ (pink) and IGFBP-5 (green) using ZDOCK 3.0.2, illustrating ten key interface residues at the predicted binding site. The top-ranked model based on ZDOCK scoring was selected for visualization. Structural analysis identified 24 residues within 3 Å, suggesting potential direct contact, as detailed in Supplementary Table S3.

Our preliminary results demonstrated a significant positive correlation between ERβ and IGFBP-5 expression in CRC (Supplementary Figure S3). Upon further stratification, we observed that this correlation was present specifically in colon cancer patient samples but not in normal tissues. In normal colonic samples, ERβ and IGFBP-5 expression showed a moderate negative correlation (*P* = 0.007, *R* = −0.41), while in colon cancer tissues, a weak but statistically significant positive correlation (*P* = 0.0065, *R* = 0.16) (Figure 8A) was identified. Interestingly, this pattern was not observed in rectal cancer tissues, where no statistically significant correlation was detected (Supplementary Figure S4).

To validate this association at the transcriptional level, ERβ expression was silenced in two colon cancer cell lines. As shown in Figure 8B, knockdown of ERβ resulted in a marked down-regulation of IGFBP-5 mRNA expression in both cell lines. This was further supported at the protein level: immunofluorescence analysis (Figure 8C) and Western blotting (Figure 8D) confirmed a significant decrease in IGFBP-5 protein expression following ERβ silencing in HCT-116 cells.

To investigate a potential association between ERβ and IGFBP-5, co-immunoprecipitation assays were performed in HCT-116 cells. As depicted in Figure 8E, IGFBP-5 was detected in the ERβ-immunoprecipitated complex, whereas no signal was observed in the IgG control, supporting the specificity of the assay. Due to the limited amount of protein recovered from the immunoprecipitation procedure, input signals were weak, and further validation using additional controls would be required to confirm the interaction. In addition, a protein–protein docking analysis was conducted to explore the potential structural basis of the association between ERβ and IGFBP-5 (Figure 8F). The top-ranked docking model, as determined by ZDOCK scoring, was selected for visualization. The predicted complex revealed multiple residues located within 3 Å of each other at the interface, suggesting possible spatial proximity between ERβ and IGFBP-5. These residues are detailed in Supplementary Table S3. The threshold of <3 Å interatomic distance is a widely accepted criterion to define direct physical contact in protein–protein interaction models [26,37]. Notably, the predicted interface residues were mapped to the ligand-binding domain region of ERβ, which may provide a structural context for the observed association. However, as docking simulations are predictive and based on rigid-body assumptions, these findings should be interpreted with caution and do not constitute definitive evidence of direct physical interaction.

In summary, these findings suggest that ERβ is associated with IGFBP-5 at both transcriptional and protein levels in colon cancer cells. While co-immunoprecipitation and *in silico* docking analyses indicate a potential interaction between the two proteins, further experimental validation will be required to confirm the nature and functional significance of this association.

### The potential detrimental role of ERβ and IGFBP-5 in colon cancer

**Figure 9:**
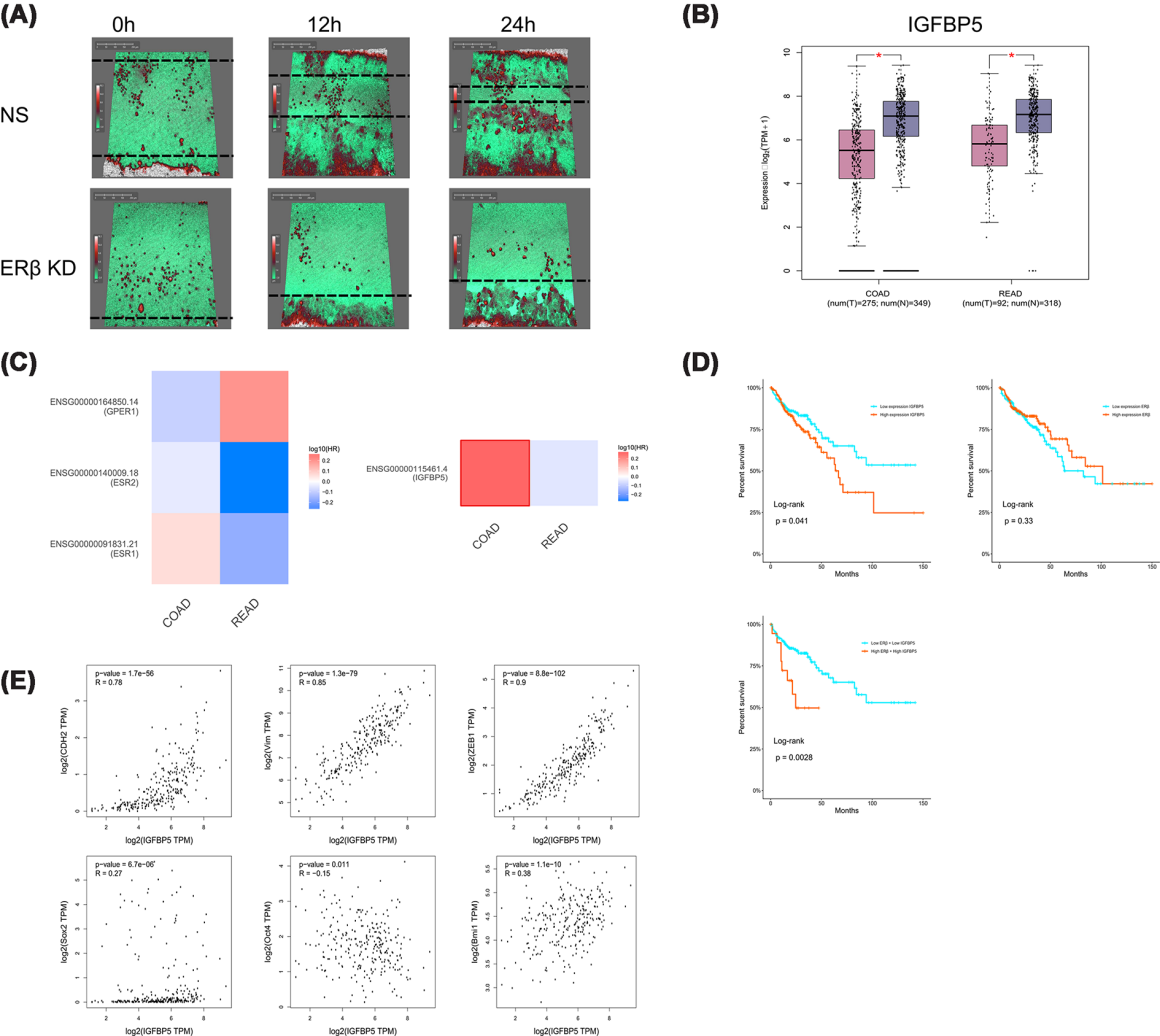
Association of ERβ and IGFBP-5 with migratory capacity and clinical outcomes in colon cancer. **(A**) The effect of ERβ silencing on wound healing in HCT-116 colon cancer cells, as assessed by the HoloMonitor live-cell imaging system. Cells were transfected with ERβ siRNA and monitored for 24 h. Red-shaded areas represent regions of cell proliferation, whereas green-shaded areas indicate regions without cellular coverage. White regions reflect areas of high cell density that exceed the detection threshold of the laser-based imaging system. Compared with the control group, ERβ-silenced cells exhibited delayed migration and incomplete closure of the wound area, suggesting impaired migratory capacity following ERβ knockdown. (**B**) Differential expression of IGFBP-5 in CRC and matched normal tissues, analysed using the GEPIA2 platform. The box plots display gene expression in log2(TPM + 1) units, comparing tumour samples with normal samples from the TCGA and GTEx databases. For COAD, the analysis included 275 tumour and 349 normal samples; for rectum adenocarcinoma (READ), 92 tumour and 318 normal samples were analysed. Purple boxes represent normal tissues, and pink boxes indicate tumour tissues. Statistical comparisons were performed using Student's *t*-test. (**C**) Prognostic landscape of IGFBP-5 and ERs (including ERβ) in colon and rectal cancer, based on TCGA data. GEPIA2-generated risk maps show the prognostic value of gene expression, with red blocks denoting poor prognosis and blue blocks indicating favourable outcomes. Statistically significant results (*P* <0.05) are outlined with a black frame. (**D**) Kaplan–Meier survival analysis based on TCGA colon adenocarcinoma database. The upper left panel shows overall survival (OS) stratified by IGFBP-5 expression. The upper right panel shows OS based on ERβ expression. The lower panel shows a two-gene survival analysis based on the combined expression of ERβ and IGFBP-5. The orange line represents high gene expression; the blue line indicates low expression. Censored data points are marked by plus signs (+). A steeper curve indicates a lower survival probability over time. (**E**) Pearson correlation analysis between IGFBP-5 and epithelial–mesenchymal transition (EMT)-related genes (CDH2/N-cadherin, VIM/vimentin, ZEB1, SOX2, Oct4, and Bmi1) in TCGA colon cancer tissues using GEPIA2. The *x*-axis represents IGFBP-5 mRNA expression levels, while the *y*-axis shows expression of individual EMT markers. Strong positive correlations were observed with N-cadherin, vimentin, and ZEB1.

Although prior studies have proposed a protective role for ERβ in CRC, our data challenge this notion. Functional experiments demonstrated that silencing ERβ significantly inhibited the proliferation of colon cancer cells. Furthermore, using the HoloMonitor-based wound healing assay, we observed that ERβ knockdown appeared to reduce cell migration. As shown in Figure 9A, while control cells nearly closed the wound after 24 h, ERβ-silenced cells exhibited a visibly reduced migration capacity, leaving a substantial gap unclosed. Time-lapse imaging from two independent experiments consistently supported this observation (Supplementary Videos S1 and S2) [38–41]. However, due to technical limitations in image acquisition, quantitative analysis of wound closure was not feasible. Therefore, these findings should be interpreted with caution. Taken together, these results indicate that ERβ may be associated with proliferative and migratory phenotypes in colon cancer cells, rather than functioning solely as a tumour suppressor.

To investigate the differential expression patterns of ERβ and IGFBP-5 in cancer *versus* normal tissues, we analysed mRNA levels using the GEPIA2 platform. Boxplot analysis revealed a significant down-regulation of IGFBP-5 in CRC tissues compared with adjacent normal controls (Figure 9B), a finding corroborated by results from our *in vitro* colon cancer model (Supplementary Figure S5).

Notably, high IGFBP-5 expression was significantly associated with poor OS in colon cancer patients (Figure 9C). Kaplan–Meier survival analysis based on TCGA data for 483 colon cancer patients demonstrated that elevated IGFBP-5 expression correlated with shorter survival times (*P* = 0.041) (Figure 9D), consistent with results from the GEPIA2 database (Figure 9C). Other IGFBPs were also examined, but only IGFBP-5 showed a substantial impact on OS in CRC patients. To further explore the combined clinical relevance of ERβ and IGFBP-5, a two-gene survival analysis was performed. Patients with concurrent high expression of both ERβ and IGFBP-5 exhibited significantly worse OS compared with those with low expression of both genes (*P* = 0.0028) (Figure 9D), suggesting a potential cooperative association in colon cancer progression. Consistent results were also observed for disease-free survival using an independent GEPIA2 dataset (*P* = 0.0081) (Supplementary Figure S6).

In contrast, while ERβ expression positively correlated with IGFBP-5 levels, it did not exhibit a statistically significant association with patient prognosis (Figure 9C,D). Interestingly, a non-significant trend toward improved survival was observed (*P* = 0.33) (Figure 9D). Additional subgroup analysis by sex did not reveal any notable survival differences when stratified by sex (data not shown).

Further correlation analysis using colon cancer transcriptomic data revealed that IGFBP-5 positively correlates with several key markers implicated in EMT and stemness. As shown in Figure 9E, strong positive correlations were observed between IGFBP-5 and N-cadherin (CDH2, *R* = 0.78), vimentin (VIM,* R* = 0.85), and ZEB1 (*R* = 0.90)—all well-established mediators of EMT. In contrast, IGFBP-5 exhibited weaker correlations with stemness-related genes including SOX2 (*R* = 0.27), Oct4 (*R* = 0.15), and Bmi1 (*R* = 0.38). These findings indicate that IGFBP-5 expression is strongly correlated with EMT-related markers, suggesting a potential association with EMT-related processes in colon cancer. To further investigate the subcellular localization of IGFBP-5, immunofluorescence staining was performed in colon cancer cells. As shown in Supplementary Figure S7, IGFBP-5 was predominantly localised in the cytoplasm and along the cell membrane, displaying a punctate and vesicle-like distribution pattern. This localization is consistent with data from the Human Protein Atlas, where IGFBP-5 is mainly detected in vesicles and is a secreted protein. Such a distribution supports a potential role for IGFBP-5 in extracellular secretion and/or cytoplasmic signalling pathways rather than nuclear processes. Together, these observations provide a possible mechanistic link between IGFBP-5 localization and its association with EMT-related processes in colon cancer.

In summary, although ERβ has traditionally been viewed as protective in CRC, our data suggest a more nuanced role. Both ERβ and IGFBP-5 may exhibit context-dependent roles in colon cancer, and under certain experimental conditions, may be associated with enhanced proliferative and migratory capacities, as well as adverse clinical outcomes in colon cancer. These results highlight the complexity of ERβ signalling and suggest that its role in colon cancer progression may depend on specific molecular and cellular contexts.

### Rapid estrogenic signalling as a potential ERβ-independent mechanism

Given the absence of significant changes in ERβ expression following 48-h treatment with either 27-OHC or E2, and considering the context-dependent role of ERβ suggested in the ‘Results’ section, we noted that morphological changes in colon cancer cells occurred rapidly, within 15–20 min after treatment initiation. This observation suggested that, in addition to transcriptional regulation mediated by nuclear receptors such as ERβ, rapid non-genomic signalling pathways might also be involved in mediating estrogenic responses. Therefore, we next investigated short-term cellular responses to E2 and 27-OHC to explore potential rapid signalling mechanisms.

**Figure 10:**
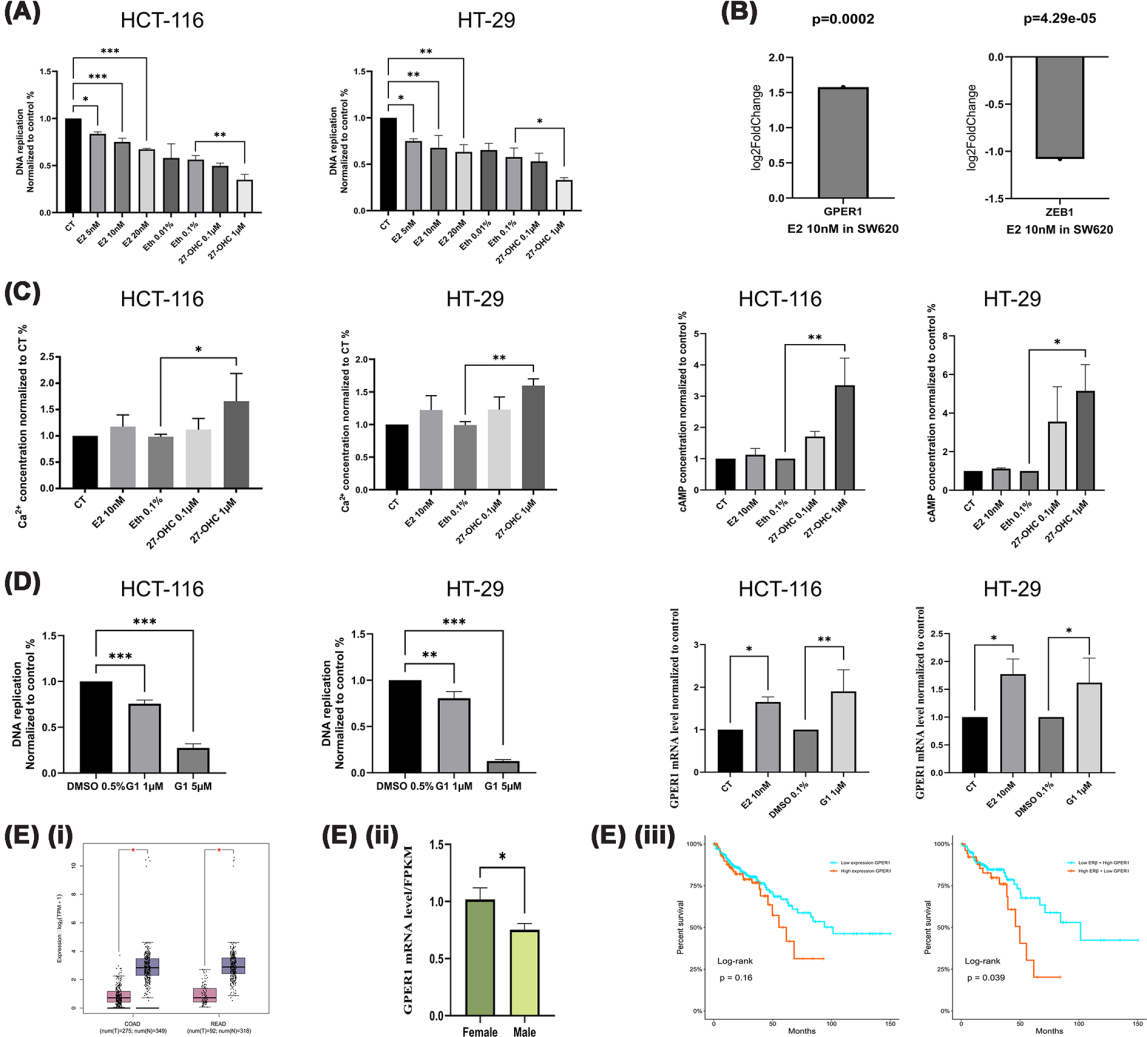
Rapid estrogenic signalling and GPER1-mediated effects in colon cancer cells. (**A**) Effects of short-term exposure to E2 and 27-OHC on cell proliferation in HT-29 colon cancer cells. Cells were treated with E2 (5, 10, and 20 nM) or 27-OHC (0.1 and 1 μM) for 15 min, followed by incubation in phenol red-free SFM. Corresponding vehicle controls included phenol red-free SFM alone and ethanol at concentrations of 0.01% and 0.1%, matching the solvent concentrations used for 27-OHC. All experiments were performed in three independent biological replicates, each in technical triplicate (*n* = 3). Proliferation data were normalised to the phenol red-free SFM control and presented as fold change. Statistical analysis was conducted using one-way ANOVA with Dunnett's *post hoc* test. Data are presented as mean ± SD. (**B**) Differential gene expression in SW620 cells treated with 10 nM E2 for 24 h based on mRNA sequencing data (GSE112568). The graphs depict up-regulation of GPER1 and down-regulation of ZEB1 following E2 treatment. Each group was analysed in duplicate (*n* = 2). (**C**) Intracellular Ca^2+^ concentrations and cAMP levels measured in HCT-116 and HT-29 cells following 15-min treatment with E2 (10 nM) or 27-OHC (0.1 and 1 μM). Control groups received phenol red-free SFM or 0.1% ethanol, respectively. All data were normalised to the SFM control and presented as fold change. Experiments were conducted in three independent biological replicates (*n* = 3), each in technical triplicate. Statistical comparisons were made using one-way ANOVA with Dunnett's *post hoc* test. Data are shown as mean ± SD. (**D**) Anti-proliferative effects of G1, a selective GPER1 agonist, following a 15-min exposure in HCT-116 and HT-29 cells. Cells were subsequently incubated in phenol red-free SFM for 48 h. Additionally, mRNA expression of GPER1 following short-term exposure to E2 and G1 was assessed. All experiments were performed in triplicate (*n* = 3), and data were normalised to vehicle controls. Statistical significance was determined using one-way ANOVA with Dunnett's *post hoc* test. Data are expressed as mean ± SD. (**E**) GPER1 expression in CRC based on TCGA and GTEx datasets. **E(i)** Differential expression of GPER1 in colorectal tumour and normal tissues analysed via GEPIA2. For COAD, 275 tumour and 349 normal samples were included; for READ, 92 tumour and 318 normal samples were analysed. Gene expression is shown in log_2_ (TPM + 1) units. Purple boxes represent normal tissues, and pink boxes represent tumour tissues. Statistical comparisons were performed using Student's *t*-test. **E(ii)** GPER1 expression levels in 438 TCGA colon cancer patients (204 females and 234 males), highlighting sex-specific expression differences. Data are shown as mean ± SD and analysed by Student's *t*-test. **E(iii)** Kaplan–Meier survival analysis of GPER1 and ERβ expression in 438 colon adenocarcinoma patients. Patients were stratified into high and low expression groups. The orange line denotes high expression and the blue line denotes low expression. Censored observations are indicated by plus signs (+). A steeper curve corresponds to lower survival probability. Statistical significance was *P* = 0.16 and *P* = 0.039.

As illustrated in Figure 10A, a 15-min exposure to E2 at concentrations ranging from 5 to 20 nM resulted in a measurable reduction in cell replication of both HCT-116 and HT-29 colon cancer cell lines compared with untreated controls. These findings indicate that even transient E2 exposure can elicit a rapid and measurable inhibitory effect on tumour cell viability. In contrast, while 0.1 μM 27-OHC induced a modest but non-significant reduction in cell proliferation, treatment with 1 μM 27-OHC significantly decreased proliferation in both cell lines, suggesting a dose-dependent short-term anti-proliferative effect.

To elucidate the molecular basis of this rapid response, we analysed transcriptomic data from the GSE112568 dataset. In E2-treated SW620 cells, GPER1 expression was markedly up-regulated, while ZEB1, a transcription factor associated with EMT, was significantly down-regulated (Figure 10B). These observations suggest that GPER1 may be involved in estrogen-responsive signalling; however, no direct causal relationship can be established based on these data alone.

Previous studies have shown that GPER1 activation rapidly triggers intracellular calcium (Ca^2+^) and cAMP signalling [42]. To evaluate whether these second messengers are involved in the short-term responses to E2 and 27-OHC, we treated HCT-116 and HT-29 cells with the compounds for 15 min and assessed changes in intracellular Ca^2+^ levels. As shown in Figure 10C, E2 induced a slight but non-significant increase in Ca^2+^ levels. However, 27-OHC led to a concentration-dependent elevation in intracellular Ca^2+^, with 1 μM 27-OHC producing a statistically significant increase compared with the ethanol control, suggesting activation of a rapid Ca^2+^ signalling cascade, potentially through GPER1.

Parallel measurements of intracellular cAMP levels further supported these findings. While E2 did not significantly alter cAMP concentrations, 27-OHC treatment induced a dose-dependent increase, with 1 μM 27-OHC significantly enhancing cAMP levels (Figure 10C). These results indicate activation of rapid intracellular signalling pathways. However, as Ca^2+^ and cAMP responses can be mediated by multiple G protein-coupled receptors, these findings do not specifically confirm GPER1 involvement.

To directly evaluate the functional role of GPER1 in mediating rapid estrogenic responses, we treated HCT-116 and HT-29 cells with **G1**, a selective GPER1 agonist, for 15 min, followed by a 48-h incubation in phenol red-free SFM. As shown in Figure 10D, G1 significantly suppressed proliferation in both cell lines in a dose-dependent manner, closely mimicking the effects of 27-OHC and E2. These findings indicate that activation of GPER1 is sufficient to induce anti-proliferative responses under the tested conditions in colon cancer cells.

To explore the clinical relevance of GPER1 expression in CRC, we analysed data from TCGA. As shown in Figure 10E, GPER1 expression was significantly reduced in tumour tissues compared with adjacent normal tissues. Furthermore, sex-stratified analysis revealed significant differences in GPER1 expression between male and female patients. However, Kaplan–Meier survival analysis did not reveal a statistically significant association between GPER1 expression levels and OS in either sex. Interestingly, combined survival analysis revealed that patients with low ERβ and high GPER1 expression exhibited significantly improved OS compared with those with high ERβ and low GPER1 expression (*P* = 0.039). This observation suggests that while GPER1 alone may have limited prognostic value, its effect may become evident in the context of ERβ expression, indicating a potential interaction between nuclear and membrane estrogen signalling pathways in CRC.

Taken together, these findings suggest that rapid estrogenic responses in colon cancer cells may involve ERβ-independent signalling pathways. While GPER1-associated signalling represents a potential contributor, the current data are insufficient to establish a direct mechanistic link. Additional exploratory transcriptomic analysis of GPER1 activation revealed alterations in metabolic pathways (Supplementary Figure S8 and Supplementary Table S4). Further studies are required to clarify the relationship between ERβ, IGFBP-5, and alternative estrogen-responsive pathways.

## Discussion

Hormone replacement therapy (HRT) has been associated with a reduced risk of CRC in postmenopausal women, and several preclinical studies have demonstrated the protective effects of estrogen [43–46]. However, results from the large-scale Women's Health Initiative trial indicated that combined hormone therapy did not confer a protective benefit against CRC and is therefore not recommended for CRC prevention or treatment [47]. Despite this controversy, accumulating evidence from both *in vitro* and *in vivo* studies continues to support the anti-tumourigenic effects of estrogen in CRC. Nevertheless, the underlying mechanisms by which estrogen mediates its effects remain insufficiently understood. This highlights the necessity of identifying appropriate molecular targets and patient subgroups that might benefit from estrogen-based interventions.

Among ERs, ERβ is primarily expressed in normal colorectal epithelial cells, with its expression markedly reduced during CRC progression [14]. This down-regulation has been associated with more aggressive tumour phenotypes, suggesting a tumour-suppressive function [48]. However, in contrast with this paradigm, our study revealed that knockdown of ERβ in HCT-116 and HT-29 colon cancer cells resulted in significant inhibition of both proliferation and migration, partially challenging the prevailing notion of ERβ's protective role. While the proliferation assays in the present study primarily reflect DNA replication activity, additional validation using complementary approaches, such as flow cytometry-based cell cycle or apoptosis analysis, would help to further clarify the underlying mechanisms. Notably, most prior studies have relied on overexpression models of ERβ, while investigations on ERβ silencing remain limited, particularly in terms of its effects on cell proliferation.

To gain further insight, we utilised SMARTpool siRNA targeting multiple ERβ isoforms (1, 2, 3, 5, and 6). We hypothesize that these isoforms may have distinct, and potentially opposing, roles in CRC. For example, in glioblastoma and triple-negative breast cancer, ERβ isoform 1 exhibits tumour-suppressive activity, whereas isoforms 2 and 5 are implicated in enhanced migration and proliferation [49,50]. Given the paucity of data on the isoform-specific roles of ERβ in CRC, further functional dissection of these variants is warranted.

In addition, our study is the first to reveal a shift in the correlation between ERβ and IGFBP-5, from negative in normal colon tissue to positive in CRC tissue. *In vitro*, silencing ERβ not only suppressed proliferation and migration in colon cancer cells but also significantly down-regulated IGFBP-5 expression. Additionally, patients with concurrent high expression of ERβ and IGFBP-5 exhibited poorer OS in colon cancer. Notably, while IGFBP-5 expression alone was associated with survival, ERβ expression alone was not significantly associated with prognosis. This suggests that the prognostic impact of ERβ may be context-dependent and influenced by co-expressed molecular partners, including IGFBP-5 and GPER1. Co-immunoprecipitation and protein docking analyses provided preliminary evidence of a direct physical interaction between ERβ and IGFBP-5. TCGA data analysis showed a robust positive correlation between IGFBP-5 and EMT markers such as N-cadherin, vimentin, and ZEB1, supporting a potential role of IGFBP-5 in CRC cell migration and invasiveness. Notably, the biological functions of IGFBP-5 are known to be highly dependent on its subcellular localization. In our study, IGFBP-5 was predominantly observed in the cytoplasm and at the cell periphery, displaying a vesicle-like distribution pattern, which is consistent with a secretory protein. This localization suggests that IGFBP-5 may primarily exert its effects through extracellular or cytoplasmic mechanisms, such as modulating IGF signalling or interacting with cell surface receptors. Furthermore, cytoplasmic IGFBP-5 has been implicated in cell migration and extracellular matrix remodelling, which is in line with our EMT-related findings. A weaker signal was occasionally observed in perinuclear regions; however, definitive nuclear localisation could not be established under the current imaging conditions.

Although IGFBP-5 has not been extensively studied in CRC, evidence from glioblastoma suggests that its knockdown reduces EMT marker expression and suppresses migration [51]. Furthermore, IGFBP-5 has been implicated in regulating proliferation and apoptosis in CRC cells [17], possibly through modulation of the WNT/β-catenin signalling pathway—an axis also regulated by ERβ [52]. Our results reinforce this link: treatment with E2 and 27-OHC reduced IGFBP-5 expression and inhibited cell proliferation and migration, and combining ERβ silencing with E2/27-OHC treatment led to further down-regulation of IGFBP-5, accompanied by increased DNA damage marker (pH2AX) and apoptotic marker (cPARP) expression. However, given that apoptosis-related processes may influence protein expression levels, it remains possible that the observed reduction in IGFBP-5 is partially influenced by treatment-induced cellular stress rather than direct regulation alone. These data suggest a potential link between ERβ and IGFBP-5 in CRC progression.

Beyond genomic effects, our study also investigated the role of GPER1, a membrane-associated receptor mediating rapid estrogen signalling. Transcriptomic analysis of E2-treated cells showed significant up-regulation of GPER1 mRNA, and HoloMonitor-based real-time imaging revealed rapid morphological changes within minutes of E2 exposure. A brief 15-min E2 treatment followed by incubation for 48 h continued to inhibit proliferation and up-regulate GPER1, suggesting that early activation of GPER1 may contribute, at least in part, to the observed long-term anti-proliferative effects. Although E2 has been reported to stimulate intracellular cAMP and Ca^2+^ release *via* GPER1, our study did not detect significant alterations in these second messengers at the 15-min timepoint. This discrepancy may be attributed to cell-type-specific and temporal variations in estrogen signalling responses, as supported by earlier studies [10,53].

To further investigate GPER1's functional role, we utilised G1, a selective GPER1 agonist, which recapitulated the anti-proliferative effects of E2 in HCT-116 and HT-29 cells. Transcriptomic profiling of G1-treated SW620 cells revealed differential gene expression patterns enriched in metabolic pathways, especially cholesterol metabolism. Notably, several of the top up-regulated genes—ABCA1, ABCG1, APOC1, IDH2, LIPC, NPC1L1, PCSK9, and TSKU—are implicated in lipid metabolism and CRC pathogenesis. Down-regulation of ZEB1 was also observed, suggesting anti-EMT activity. These findings are consistent with prior studies indicating that GPER1 activation helps maintain metabolic homeostasis, mitigate obesity-related inflammation, and prevent insulin resistance [54]. Conversely, GPER1 deficiency has been linked to increased adiposity and systemic inflammation in murine models, suggesting a potential mechanistic link between obesity and CRC *via* GPER1.

Nonetheless, the role of GPER1 in CRC remains controversial. Bustos et al*.* demonstrated that GPER1 exerts anti-proliferative effects under normoxic conditions but may promote migration and survival under hypoxia [55]. Moreover, our TCGA analysis highlighted sexually dimorphic expression of GPER1, with significantly higher expression in female patients. Prior reports also suggest a sex-specific prognostic relevance, wherein elevated GPER1 expression is associated with poorer outcomes in late-stage female CRC patients but not in males. Collectively, these findings point to a complex, context-dependent role of GPER1 in CRC.

**Figure 11:**
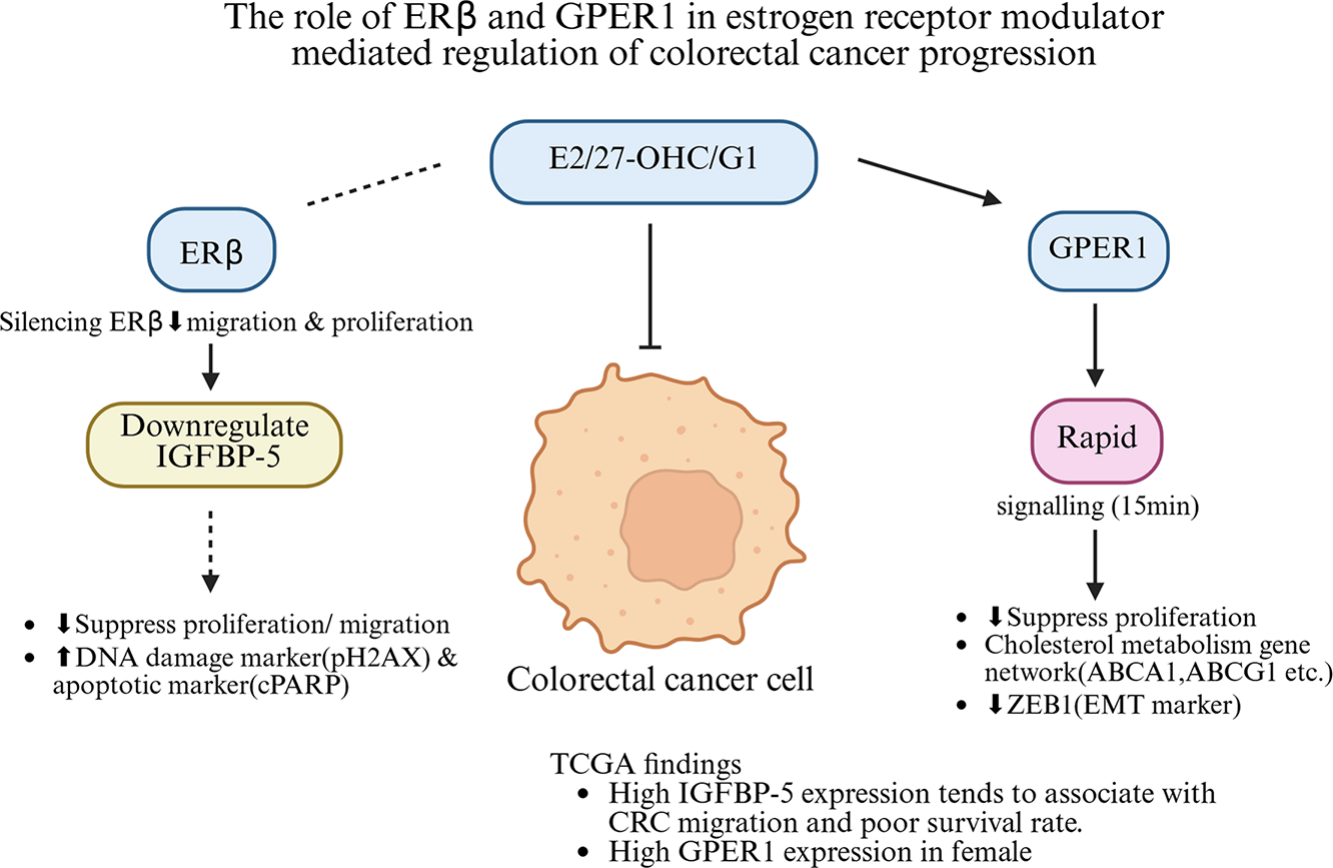
Proposed model of ERβ- and GPER1-associated estrogen signalling pathways in colon cancer. A schematic to summarise the role of ERβ and GPER1 following exposure to 27-OHC, E2, or G1. The changing of ERβ and GPER1 leads to distinct downstream effects, including the down-regulation of IGFBP-5 and rapid non-genomic signalling, respectively. The *dotted arrows* indicate interactions or regulatory effects for which direct experimental evidence is currently lacking in the present study. Specifically, although silencing ERβ and treatment with E2 or 27-OHC results in down-regulation of IGFBP-5 and phenotypic alterations in CRC cells, a **c***ausal mechanistic link* between ERβ activation and SERMs treatment has not been conclusively demonstrated. Moreover, the downstream effect of IGFBP-5 reduction on cell phenotypes is inferred based on correlation analysis and previous literature, but requires further validation. This schematic integrates current findings with working hypotheses to provide a conceptual framework for ERs signalling in CRC.

In conclusion, as shown in Figure 11, our study demonstrates that ERβ may not function exclusively as a tumour suppressor in CRC, and its role may vary depending on the molecular context, including its interaction with IGFBP-5 and GPER1-associated signalling pathways. Estrogen signalling in CRC may involve both ERβ-dependent and ERβ-independent pathways. We report, to our knowledge, a potential physical interaction and an association with poorer survival between ERβ and IGFBP-5, suggesting a potential relationship between ERβ and IGFBP-5 in CRC progression. In contrast, GPER1 may represent a potential contributor to rapid, non-genomic estrogen responses that inhibit CRC cell proliferation. Short-term activation of GPER1 using 27-OHC, E2, or G1 yields sustained anti-proliferative effects and modulates key metabolic and EMT-associated pathways. The ERβ/IGFBP-5 axis and GPER1 related pathway are not mutually exclusive and may operate in parallel or under different cellular contexts. Such complexity may partly explain the heterogeneous responses to estrogen observed across different colon cancer models. Notably, GPER1 may also link obesity-related dysregulation to CRC development, expanding its potential as a therapeutic target. Compared with ERβ-dependent signalling, GPER1 activation may represent an alternative pathway for leveraging estrogen signalling in CRC therapy.

## Clinical perspectives

- *Background as to why the study was undertaken*: Obesity contributes to CRC by elevating levels of estrogen, 27-OHC, and IGFs and yet HRT reduces CRC incidence and mortality, suggesting a protective role for estrogen. The present study aimed to unravel the roles of ERs, ERβ, and GPER1, in CRC progression and identify associations with the IGF signalling axis.
- *A brief summary of the results*: A strong positive correlation between ERβ and IGFBP-5 in CRC tissues was discovered, and high IGFBP-5 expression was significantly associated with poor patient outcomes. Co-immunoprecipitation confirmed a direct interaction between ERβ and IGFBP-5. Silencing ERβ, counterintuitively, inhibited colon cancer cell proliferation and migration, and was accompanied by a marked reduction in IGFBP-5. Both estrogen and 27-OHC suppressed CRC cell proliferation and IGFBP-5 expression, which was mediated by GPER1.
- *The potential significance of the results to human health and disease*: IGFBP-5 may act as a potential prognostic marker with GPER1 as a promising therapeutic target.

## Supplementary Material

Supplementary Figures S1-S8 and Tables S1-S4

Supplementary Excel Data S1

Supplementary Video S1

Supplementary Video S1

Supplementary Video S2

Supplementary Video S2

## Data Availability

Data sharing is not applicable to the paper, or all supporting data are included within the main article and its supplementary files

## Open Access

Open access for this article was enabled by the participation of the University of Bristol in an all-inclusive Read & Publish agreement with Portland Press and the Biochemical Society under an agreement with JISC.

## Abbreviations

27-OHC: 27-hydroxycholesterol
ANOVA: Analysis of variance
BCA: Bicinchoninic acid
cAMP: cyclic adenosine monophosphate
CCLE: Cancer Cell Line Encyclopedia
COAD: colon adenocarcinoma
cPARP: cleaved poly(ADP-ribose) polymerase
CRC: colorectal cancer
DAPI: 4′,6-diamidino-2-phenylindole
DEGs: differentially expressed genes
EGF: Epidermal growth factor
EMT: epithelial–mesenchymal transition
ERs: estrogen receptors
FBS: fetal bovine serum
GAPDH: Glyceraldehyde-3-phosphate dehydrogenase
GPER1: G protein-coupled estrogen receptor 1
HRP: Horseradish peroxidase
HRT: hormone replacement therapy
IGFs: insulin-like growth factors
IGFBP-5: insulin-like growth factor-binding protein-5
OS: overall survival
pH2AX: phosphorylated histone H2AX
READ: rectum adenocarcinoma
RIPA: Radioimmunoprecipitation assay
SD: standard deviation
SERMs: selective estrogen receptor modulators
SFM: serum-free medium
STR: Short tandem repeat
TCGA: The Cancer Genome Atlas
