# Supplementary material for: Unravelling the effects of selective estrogen receptor modulators on colorectal cancer: a prognostic role for insulin-like growth factor binding protein-5
Source: Clin Sci (Lond). 2026 May 21;140(6):1011–32. doi: 10.1042/CS20258451 (PMC13199839; doi:10.1042/CS20258451)
Supplement: Supplementary Figures S1-S8 and Tables S1-S4 [file CS-2025-8451_supp.pdf]

Supplementary data

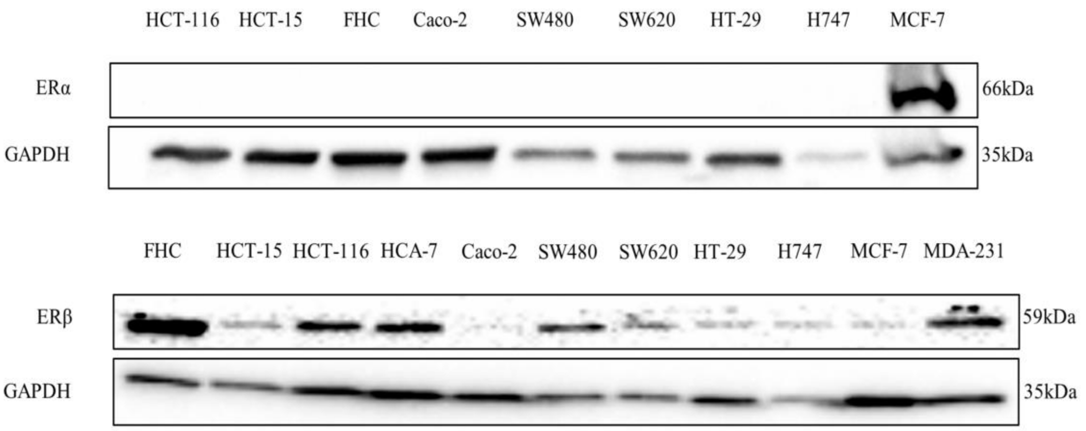

Supplementary Figure S1: Expression of ERα and ERβ in selected colorectal cancer cell lines panel through Western Blot (n=1). MCF-7 being as ERα positive control and MDA-231 being as ERβ positive control.

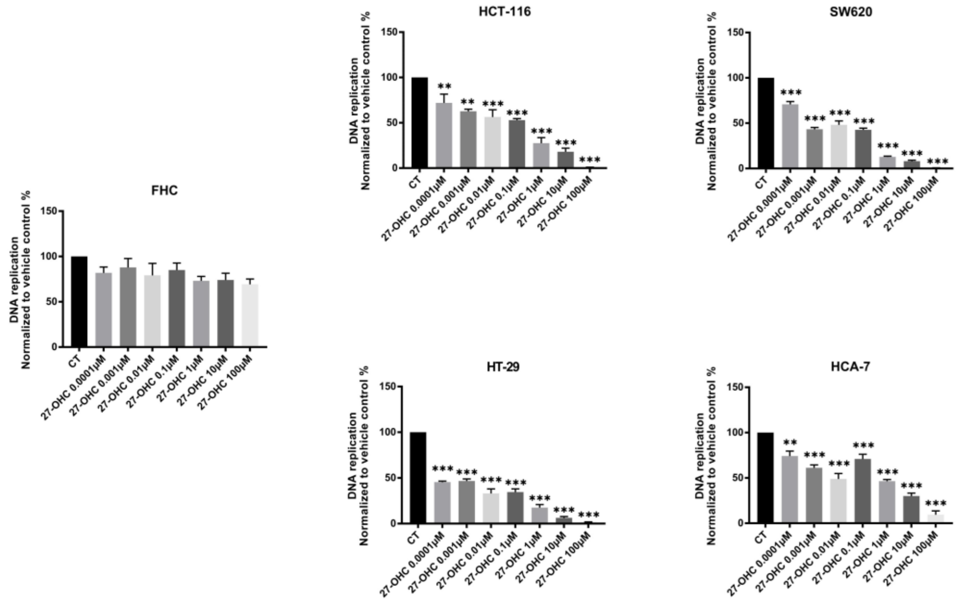

Supplementary Figure S2: Effect of 27-OHC 48 hours treatment on the proliferation of five colon cancer cell lines. This experiment was performed in triplicate and repeated three biological times (n=3). The graphs illustrate the proliferative response of five distinct colon cancer cell lines to various concentrations of 27-OHC. The x-axis indicates the different concentrations of 27-OHC, while the y-axis represents the normalized cell proliferation capacity relative to the vehicle control group. Statistical analysis of differences was conducted using a one-way ANOVA with Dunnett post-hoc test showing with mean±SD.

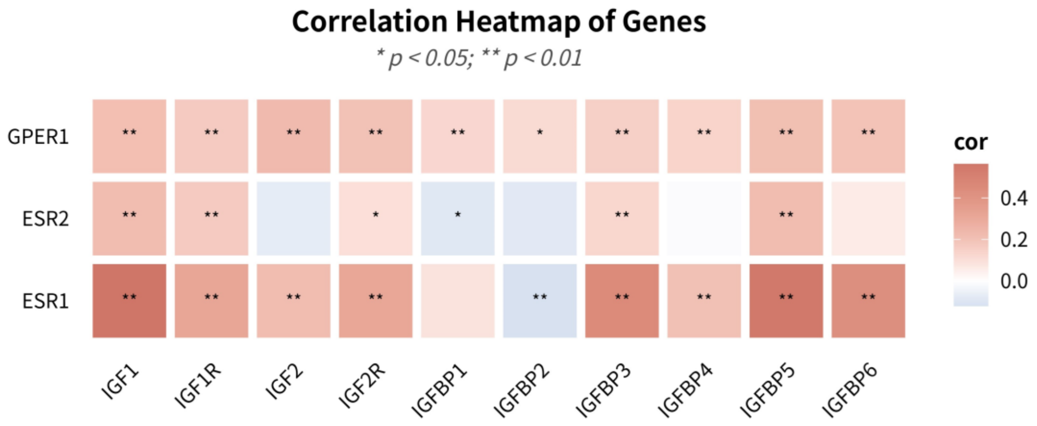

Supplementary Figure S3: Correlation analysis between estrogen receptors and the IGF axis in CRC (n=526). The figure illustrates a Spearman correlation analysis, displayed as a heatmap. ESR1 indicates ERα, ESR2 indicates ERβ. Statistical significance is indicated by a p-value of less than 0.05, where * denotes $P < 0.05$ and ** denotes $P < 0.01$. The color scheme used in the figure indicates the direction and strength of the correlations: Red signifies a positive correlation, and blue indicates a negative correlation. The intensity of the color corresponds to the strength of the relationship between the genes, with darker shades representing stronger correlations. The **pheatmap** from R package was utilized to perform correlation analyses and generate heatmaps.

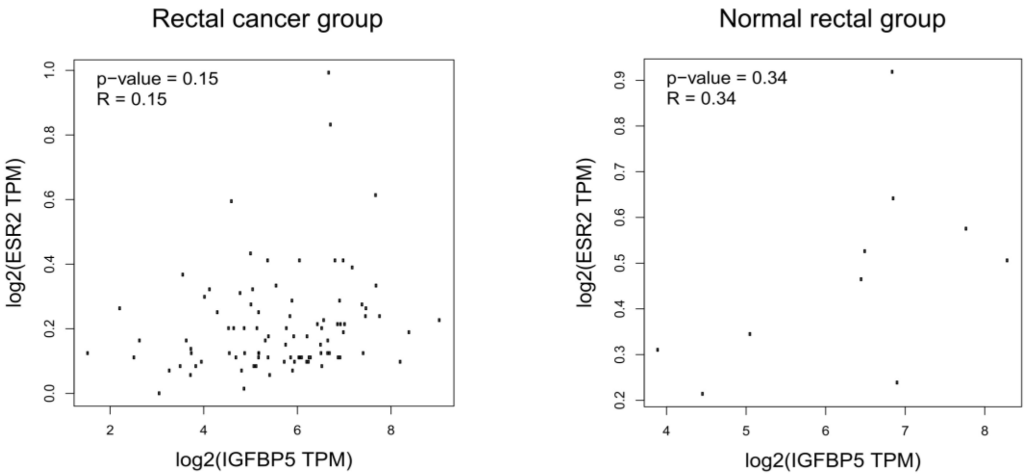

Supplementary Figure S4: Pearson correlation analysis between ERβ and IGFBP-5 mRNA expression levels in rectal cancer and normal tissues using the GEPIA2 database. The x-axis represents IGFBP-5 mRNA expression, and the y-axis represents ERβ mRNA expression. A positive correlation suggests simultaneous increases or decreases in expression of both genes, whereas a negative correlation indicates inverse expression trends. The correlation coefficient (R) ranges from −1 to 1, with values closer to ±1 indicating a stronger correlation. No statistically significant correlation was noted in rectal cancer tissues.

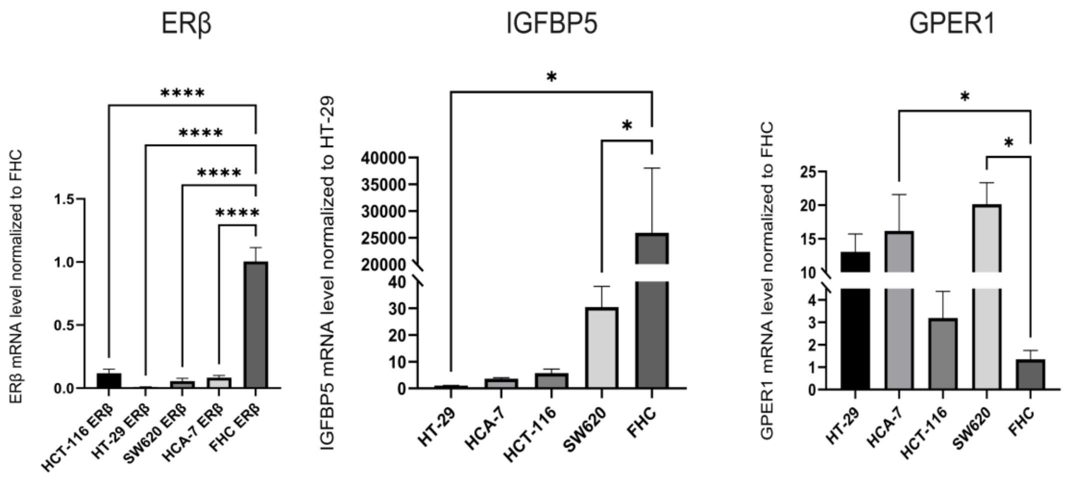

Supplementary Figure S5: Quantitative assessment of mRNA levels for IGFBP-5, ERβ, and GPER1 across four selected colon cancer cell lines, compared to a normal colon cell line. In each qRT-PCR dataset, the cell line with the lowest gene expression was designated as the control group. Statistical analysis of differences was conducted using a one-way ANOVA with Dunnett post-hoc test showing with mean±SD. This experiment was performed by three biological repeats in triplicate (n=3)

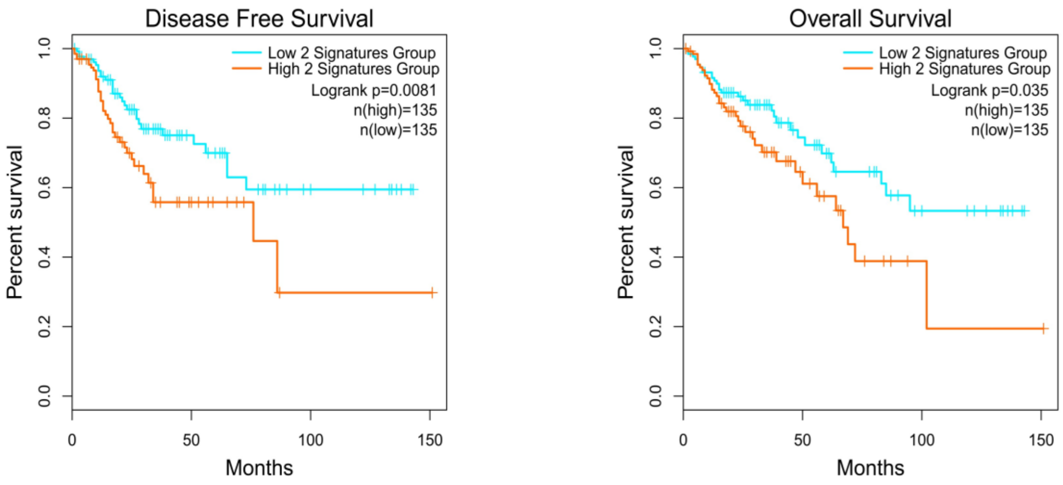

Supplementary Figure S6: Disease-free survival (DFS) analysis was conducted using the GEPIA2 platform based on TCGA colon adenocarcinoma (COAD) data. This analysis was performed using an independent platform from that used in Figure 9D and was included to complement the overall survival (OS) results. Patients were stratified according to the combined expression pattern of ERβ and IGFBP-5 using median expression values as cutoffs. Patients with concurrently high expression of both genes were defined as the high 2 Signature group, whereas those with low expression of both genes were defined as the low 2 Signatures group. Survival differences were evaluated using the log-rank test.

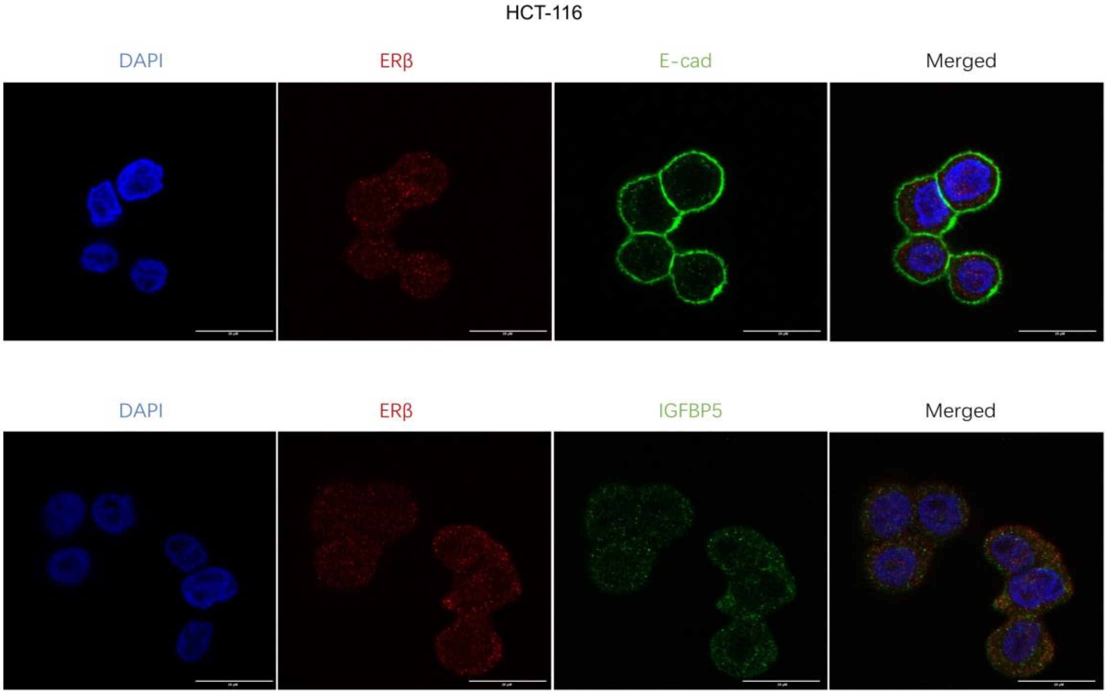

Supplementary Figure S7: Localization of ERβ and IGFBP5 in HCT-116 cell lines. Confocal microscopy reveals the intracellular localization of E-cadherin and ERβ within HCT-116 cells. E-cadherin is visualized with green fluorescence, marking its presence in the cell membrane, while ERβ is detected with red fluorescence, indicating its distribution is located in the cytoplasm. ERβ is marked by red fluorescence, IGFBP5 by green fluorescence. The nucleus is highlighted with DAPI staining, shown in blue, to provide a reference for the spatial orientation of ERβ and E-cadherin within the cells.

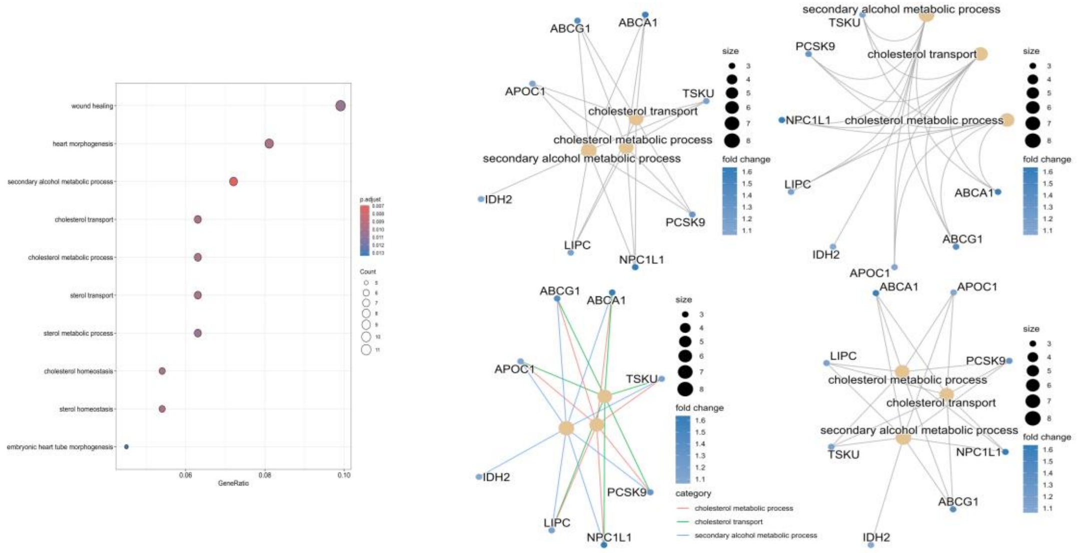

Supplementary Figure S8: Transcriptomic profiling of SW620 cells treated with G1, based on mRNA sequencing (GSE112568, n = 2). Gene Ontology (GO) enrichment analysis showing biological processes significantly modulated by G1. Circle size corresponds to the number of genes within each GO term, and colour intensity (blue to red) reflects increasing statistical significance. Signalling pathway network analysis identifying the top three enriched pathways related to G1-regulated genes. Key genes involved include ABCA1, ABCG1, APOC1, IDH2, LIPC, NPC1L1, PCSK9, and TSKU, which are implicated in cholesterol transport, sterol metabolism, and secondary alcohol processing. Functional annotations are summarized in **Supplementary Table S4.**

Supplementary Table S1: List of primer sequences for qRT-PCR

Primer	Sequence (5'to 3')
ERα	Forward 5' -GCTACGAAGTGGGAATGATGAAAG<br>Reverse 5' -TCTGGCGCTTGTGTTTCAAC
ERβ	Forward 5' -AAGTTGGCCGACAAGGAGTT<br>Reverse 5' -ACAGGCTGAGCTCCACAAAG
IGFBP-5	Forward 5' -ACAAGAGAAGCAGTGCAAACC<br>Reverse 5' -CGTCAACGTACTCCATGCCT
GPER1	Forward 5' - TGTACTTCATCAACCTGGC<br>Reverse 5' - AGGAAGAAGACGCTGCTGTA
GAPDH	Forward 5' -CATCTTCTTTTGCGTCGCCA<br>Reverse 5' -TTAAAAGCAGCCCTGGTGACC

Supplementary Table S2: The list of primary antibodies.

Protein	Molecular weight	Company	Concentration	Secondary antibody
ERα	66KDa	Santa Cruz sc-73479	1:1000	Mouse
ERβ	59KDa	Invitrogen PPZ0506	1:500	Mouse
IGFBP-5	35KDa	GroPep Bioreagents	1:500	Rabbit
pH2AX	15KDa	Abcam ab26350	1:1000	Mouse
cPARP	95KDa	BD pharmingen Asp214	1:1000	Mouse
EGFR	180KDa	Cell signalling XP(R) D38B1	1:1000	Rabbit
pEGFR	180KDa	Cell signalling T669	1:1000	Rabbit
AKT	56KDa	Cell signalling 9272S	1:1000	Rabbit
pAKT	56KDa	Cell signalling XP(R) S473	1:1000	Rabbit
α-Tubulin	51KDa	EMD Millipore DM1A	1:5000	Mouse
GAPDH	35KDa	EMD Millipore MAB274	1:5000	Mouse

Supplementary Table S3: The table shows the residues from ERβ that are located within 3 Å of IGFBP-5 after ZDOCK-based protein–protein docking. Atom count refers to the number of atoms from each residue that fall within the 3 Å interface threshold, indicating potential physical interactions.

No.	Chain	Residue ID	Residue Name	Atom Count (within 3 Å)
1	X	324	LEU (Leucine)	1
2	X	325	PHE (Phenylalanine)	13
3	X	328	VAL (Valine)	2
4	X	332	GLU (Glutamic acid)	1
5	X	413	LEU (Leucine)	1
6	X	414	VAL (Valine)	2
7	X	415	THR (Threonine)	2
8	X	416	ALA (Alanine)	2

Supplementary Table S4: Upregulated genes following G1 treatment in colon cancer

Genes name	Function
ABCA1	Cholesterol efflux
ABCG1	Cholesterol efflux
APOC1	Regulation of lipid metabolism
IDH2	Intermediary metabolism and energy production
LIPC	Triglyceride hydrolase
NPC1L1	Cholesterol absorption
PCSK9	Degradation of low-density lipoprotein receptor (LDLR)
TSKU	Cholesterol efflux, negative regulation of WNT pathway

Supplementary Video S1–S2: Time-lapse imaging of wound healing assays in control and ERβ knockdown HCT-116 colon cancer cells in 24 hours. Representative recordings from two independent experiments are shown.
